# A dual-targeting mRNA lipid nanoparticle restores ABCA1-dependent cholesterol homeostasis to promote repair of age-related bone defects

**DOI:** 10.1016/j.bioactmat.2026.08.017

**Published:** 2026-08-20

**Authors:** Mengze Cheng, Kun Xi, Zhengxia Ni, Lingjun Wang, Jincheng Tang, Wei Wang, Ziang Li, Liang Zhou, Xinzhao Jiang, Jie Wu, Qiangqiang Guo, Yiwei Zhu, Zhengjie Tao, Tianyi Li, Changdong Cui, Wenguo Cui, Liang Chen, Yong Gu

**Affiliations:** a Department of Orthopedics, The First Affiliated Hospital of Soochow University, Orthopedic Institute, Soochow University, 188 Shizi Road, Suzhou, Jiangsu, China; b Department of Orthopaedics, Shanghai Key Laboratory for Prevention and Treatment of Bone and Joint Diseases, Shanghai Institute of Traumatology and Orthopaedics, Ruijin Hospital, Shanghai Jiao Tong University School of Medicine, Shanghai, 200025, China

**Keywords:** Autophagy, Senescent bone repair, Palmitoylation, Cholesterol metabolism

## Abstract

Cholesterol regulates autophagy and aging, yet its role in bone marrow mesenchymal stem cell (BMSC) senescence remains unclear. We demonstrate that ABCA1 palmitoylation is associated with its lysosomal localization and lysosome-associated cholesterol transport, thereby contributing to autophagy inhibition, senescence, and impaired osteogenesis. However, directly perturbing ABCA1 palmitoylation impaired cholesterol efflux through plasma membrane-localized ABCA1, increased cellular cholesterol accumulation, and inhibited osteogenesis. To selectively depalmitoylate lysosomal ABCA1, we engineered a lysosome-targeted depalmitoylase, RAB7-APT2 (RA), by fusing RAB7 to APT2, the primary depalmitoylase of ABCA1. Aging-cell-targeted low-inflammatory liposomes were synthesized to deliver RA mRNA, yielding dual-targeted lipid nanoparticle (LNP@A). LNP@A selectively modulated lysosomal ABCA1 localization, reduced lysosomal cholesterol accumulation, restored autophagy, and alleviated senescence in aged BMSCs. Consequently, LNP@A enhanced osteogenesis in vitro and improved bone regeneration in an aged rat defect model. These findings reveal a role for ABCA1 spatial regulation in BMSC aging and provide a targeted strategy for age-related bone regeneration.

## Highlights

• Palmitoylation-driven cholesterol dysregulation induces BMSC senescence.
• Lysosome-targeted depalmitoylases act as a driving unit for relocalization.
• Senescence-targeted liposomes efficiently deliver the driving unit.
• Nanomotors restore cholesterol homeostasis and promote bone regeneration.

## Graphical abstract

**Figure:**
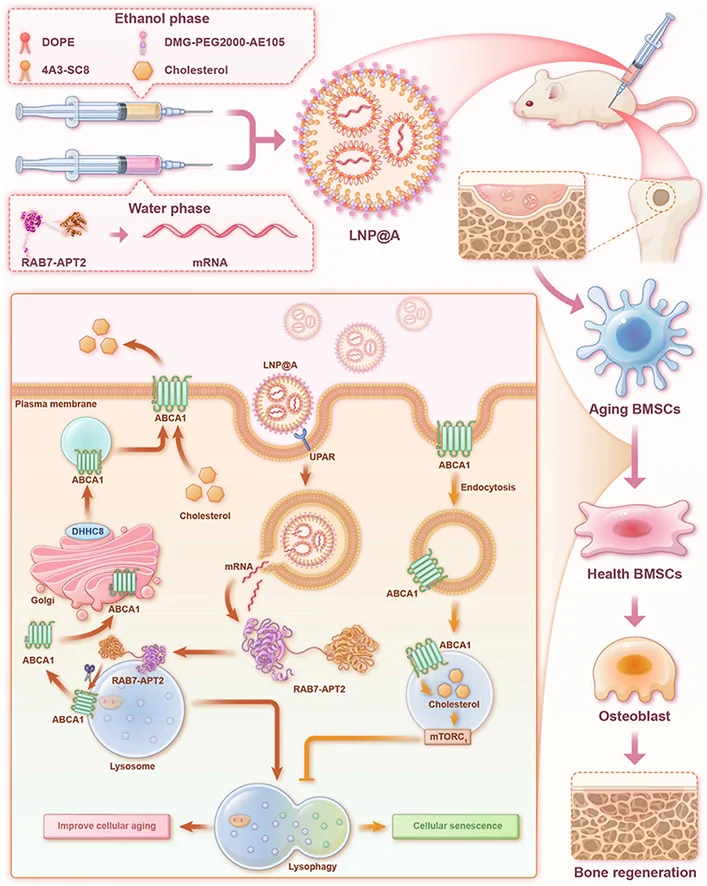

## 1 Introduction

The global population aged 65 and above is projected to reach 1.5 billion by 2025 [1], making age-related osteoporosis and non-union of bone defects significant public health concerns [2,3]. Cholesterol, a nutrient with far-reaching effects, is a crucial component of the plasma membrane, regulating membrane fluidity and the formation of microdomains [4,5]. Cholesterol and cholesterol derivatives modulate cellular senescence via various mechanisms [[6], [7], [8], [9], [10]]. Among these, cholesterol-mediated regulation of autophagy is particularly noteworthy. Cholesterol supports mTORC1 complex activity via the transmembrane proteins SLC38A9 and lycosamine [11,12]. Under nutritional abundance or aging conditions, mTORC1 maintains high activity and suppresses autophagy [13]. Additionally, cholesterol depletion has been shown to initiate autophagy in various studies [[14], [15], [16]]. Moreover, in Alzheimer's disease models, cholesterol impairs the fusion of Aβ-rich autophagosomes with lysosomes, thereby inhibiting their clearance [17,18].Previous studies showed that aging BMSCs exhibit reduced autophagy levels [19]. Restoring autophagy enhances the osteogenic potential of BMSCs by mitigating cellular senescence [20,21], whereas inhibiting autophagy accelerates cellular senescence, ultimately impairing the osteogenic differentiation of BMSCs [22]. Therefore, clarifying the role of cholesterol in autophagy suppression in aged BMSCs remains essential.

Cellular metabolism is the foundation of all biological activity [23]. The metabolic state of senescent cells exhibits multiple alterations to support phenotypic changes, such as cellular hypertrophy and senescence-associated secretory phenotype (SASP) [[24], [25], [26]]. Substantial evidence indicates that the homeostasis of cholesterol metabolism undergoes alterations during the aging process, is involved in cellular senescence, and ultimately influences the progression of age-related diseases [8,10,26]. Osteoporotic rats exhibit reduced bone mineral density (BMD) along with elevated cholesterol levels [27]. High cholesterol levels cause bone marrow mesenchymal stem cells (BMSCs) to switch from osteogenic to adipogenic differentiation [[28], [29], [30]]. Homeostasis of the cholesterol metabolism relies on the tight coordination of four dynamic modules: synthesis, absorption, conversion, and transport [10]. Numerous proteins, including NPC1 and NPC2, and members of the ABC transporter family, participate in this process [10]. Additionally, palmitoylation is a reversible post-translational modification that frequently influences the localization of membrane proteins and their interactions with biological membranes [31]. Certain cholesterol transporters that must localize to the plasma membrane and endomembrane systems to perform their transport functions rely on palmitoylation to regulate cholesterol homeostasis [[32], [33], [34]]. The ABC transporter ABCG1 relies on palmitoylation for its localization to the plasma membrane, and promotes osteogenic differentiation by influencing the Wnt pathway [35]. Although the effects of cholesterol metabolism and its transporters on bone homeostasis have been partially elucidated, whether cholesterol metabolism participates in the decline of autophagy in aged BMSCs remains unknown. Furthermore, the underlying mechanisms and transporters involved are unclear. Consequently, new treatment strategies targeting autophagy can be developed by precisely targeting palmitoylation to restore the osteogenic potential of BMSCs, ultimately promoting the healing of age-related bone defects.

Numerous studies indicate that protein palmitoylation represents a potential therapeutic target for diverse diseases [31]. Recent research suggests that interfering with GPX4 palmitoylation offers a viable strategy for regulating ferroptosis [36]. Furthermore, one study explored the application of the broad-spectrum palmitoylation inhibitor 2-bromohexadecanoic acid (2-BP) in cancer therapy [37]. However, conventional palmitoylation inhibitors such as 2-BP exert broad, non-specific effects, leading to off-target consequences that may disrupt physiological palmitoylation in healthy cells [38,39]. Moreover, certain proteins undergo Golgi palmitoylation before trafficking to multiple organelles [40,41]. If their functions differ across these organelles, direct intervention at the palmitoylation level lacks the required precision. Therefore, the development of more targeted palmitoylation-modulating strategies holds significant promise for regulating palmitoylation-associated pathological processes.

**Scheme 1:**
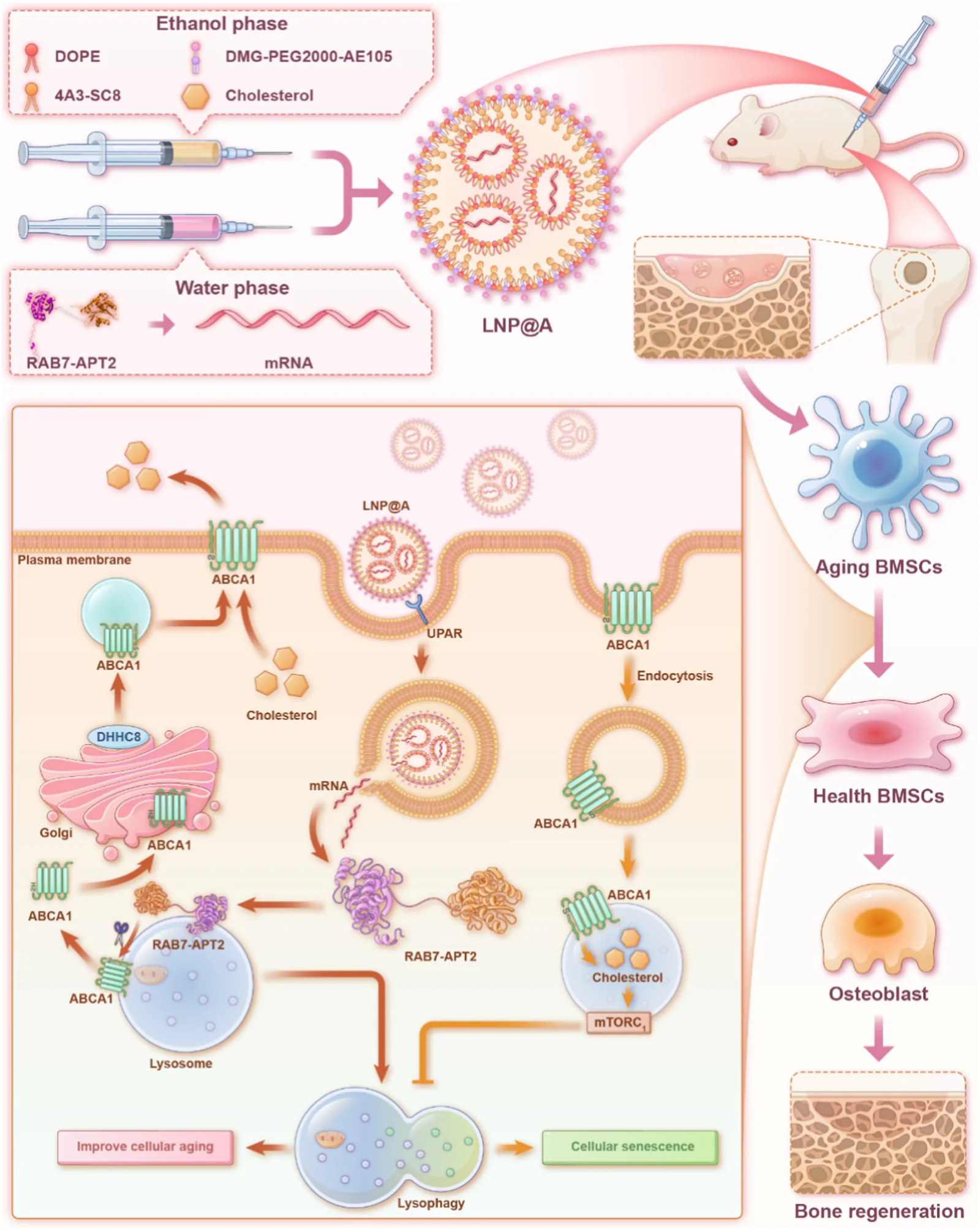
ABCA1 undergoes palmitoylation in the Golgi apparatus for transport to the plasma membrane, then enters endosomes and lysosomes via endocytosis, where it functions as a lysosomal cholesterol importer to activate mTORC1 and inhibit autophagy; depalmitoylation releases ABCA1 from the plasma membrane, endosomes, and lysosomes, allowing it to return to the Golgi apparatus for re-palmitoylation. The targeted liposome LNP@A, loaded with RAB7-APT2 engineered enzyme (RA) mRNA, shows minimal inflammatory responses. It tilts the ABCA1 palmitoylation cycle toward the plasma membrane, maintaining cellular cholesterol efflux while inhibiting lysosomal mTORC1 activation and restoring autophagy.

In this study, we aimed to address the research gap in the cholesterol metabolic crosstalk pattern among organelles in BMSCs within age-related bone defects. First, we combined transcriptome sequencing with validation experiments to establish a phenotypic correlation between cholesterol metabolism disorders and abnormal autophagy in aged BMSCs. Our findings support a model in which ABCA1 is palmitoylated by DHHC8 in the Golgi apparatus and subsequently trafficked to the plasma membrane. Following endocytosis, ABCA1 is redirected to the endolysosomal membrane, where it may deliver cholesterol to lysosomes, thereby supporting mTORC1 activation. We further identified APT2 as the major depalmitoylase of ABCA1. As a broadly distributed cytosolic depalmitoylase, APT2 may reduce the membrane association of ABCA1 through depalmitoylation, thereby facilitating its recycling to the Golgi apparatus. Conventional strategies for modulating palmitoylation lack the precision to simultaneously reverse mTORC1-mediated autophagy inhibition while preserving plasma membrane ABCA1-mediated cholesterol efflux. Accordingly, we engineered the fusion protein RA, in which RAB7 confers lysosomal targeting and APT2 mediates depalmitoylation. By encapsulating RA mRNA in a senescence-targeting lipid nanoparticle, we established the dual-targeting mRNA delivery system LNP@A. Leveraging its dual-targeting capability, LNP@A selectively intervenes in the lysosomal branch of the ABCA1 palmitoylation cycle in senescent BMSCs, while minimally perturbing physiological palmitoylation in healthy cells, thus overcoming the nonspecificity of conventional approaches. By redirecting ABCA1 to the plasma membrane, it coordinates subcellular organelle cascades, synergistically reprograms cholesterol metabolism, and enhances autophagy. This integrated regulation restores the osteogenic potential of aged BMSCs and redirects ABCA1-mediated cholesterol transport toward a youthful state. Finally, we further validated the ability of LNP@A to regulate disrupted cholesterol homeostasis, rescue the osteogenic potential of aged BMSCs, and promote the repair of age-related bone defects in a femoral condylar defect model of aged rats, providing insights into its functional and biological mechanisms. Thus, our study established an integrated therapeutic strategy for repairing age-related bone defects through a three-pronged approach: clarifying the nested cholesterol metabolism-autophagy decline phenotype, uncovering the pathological molecular mechanism of the ABCA1 palmitoylation cycle between organelles, and developing targeted nanomaterials (Scheme 1).

## 2 Results and discussion

### 2.1 Elevated cholesterol levels in aged BMSCs exacerbate cellular senescence by inhibiting autophagy

**Fig. 1:**
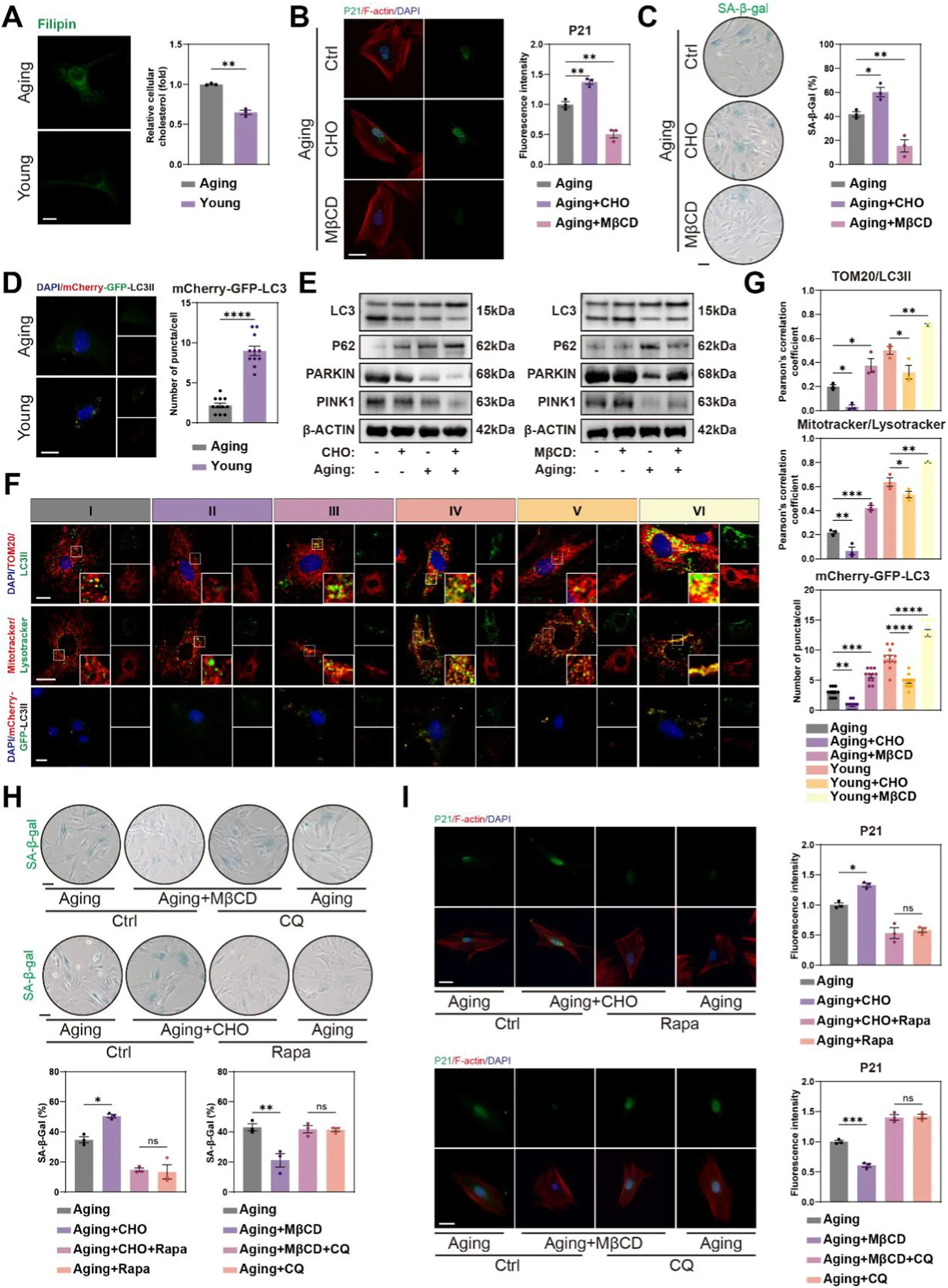
Elevated cholesterol levels in aged BMSCs exacerbate cellular senescence by inhibiting autophagy. **A.** Representative images of Filipin staining in young and aged BMSCs (scale bar = 10 μm) and intracellular cholesterol levels measured by enzymatic assay (n = 3 biologically independent samples). **B.** Representative images of P21 staining in aged BMSCs following cholesterol depletion or stimulation (scale bar = 10 μm) and semi-quantitative analysis (n = 3 biologically independent samples). **C.** Representative SA-β-gal staining images (scale bar = 20 μm) and quantitative analysis (n = 3 biologically independent samples) of cholesterol depletion or stimulation in aged BMSCs. **D.** Representative confocal images of autophagy bodies in young or aged BMSCs labeled with mCherry-GFP-LC3 (scale bar = 10 μm) and semi-quantitative analysis of autophagosomes in young or aged BMSCs labeled with mCherry-GFP-LC3, counted by participants unaware of experimental conditions (n = 11 independent biological samples). **E.** Western blot analysis of autophagy-related markers in aged BMSCs following cholesterol stimulation or depletion. **F.** Representative confocal images of LC3II and TOM20 staining in aged BMSCs following cholesterol stimulation or depletion, alongside representative confocal images of mCherry-GFP-LC3-labeled autophagosomes and Lysotracker/Mitotracker staining (scale bar = 10 μm). **G.** Semi-quantitative analysis was performed by participants unaware of experimental conditions to count autophagosomes (n = 11 biologically independent samples); perform co-localization analysis using ImageJ (n = 3 biologically independent samples). **H.** Representative SA-β-gal staining images (scale bar = 20 μm) and quantitative analysis (n = 3 biologically independent samples) following cholesterol stimulation or depletion in aged BMSCs. **I.** Representative images of P21 staining (scale bar = 10 μm) and semi-quantitative analysis (n = 3 biologically independent samples) in aged BMSCs following cholesterol stimulation or depletion. Data are presented as mean ± standard error of the mean (SEM). Statistical analysis was performed using two-tailed Student's t-test for comparisons between two groups and one-way analysis of variance (ANOVA) followed by Tukey's multiple comparisons test for comparisons among multiple groups. The number of biological replicates (n) is indicated in each figure legend. Statistical significance is indicated as *p < 0.05, **p < 0.01, and ***p < 0.001. BMSCs: Bone marrow-derived mesenchymal stem cells; MβCD and CHO represent cholesterol depletion and cholesterol stimulation, respectively; Rapa: Rapamycin; CQ: Chloroquine; SA-β-Gal: Senescence-associated β-galactosidase.

Preliminary research has indicated that cholesterol metabolism is involved in cellular aging [8,10,26]. However, whether cholesterol metabolism acts as a cause or consequence of aging process remains a long-standing debate [4]. Therefore, we directly assessed the intracellular cholesterol levels in BMSCs to investigate the role of cholesterol in BMSC senescence. Filipin staining and enzymatic assays revealed that senescent BMSCs exhibited significantly higher cholesterol levels than young BMSCs (Fig. 1A). Subsequently, we explored the effect of this alteration in cholesterol levels on BMSC senescence. Depletion of intracellular cholesterol using methyl-β-cyclodextrin (MβCD) reduced p21 and p53 expression in aged BMSCs, whereas cholesterol stimulation increased p21 and p53 expression (Fig. 1B and Supplementary Fig. 1A). Similar results were observed when detecting age-associated β-galactosidase (SA-β-gal) (Fig. 1C). We then assessed IL-1β and IL-6 expression levels via qRT-PCR to evaluate the impact of cholesterol on the senescence-associated secretory pattern (SASP) (Supplementary Fig. 1B). MβCD reduced IL-1β and IL-6 expression, whereas cholesterol stimulation produced the opposite effect. These findings indicated that elevated cholesterol levels in senescent BMSCs exacerbate cellular aging.

Extensive evidence has indicated that senescent cells frequently exhibit suppressed autophagy [[42], [43], [44], [45]], and cholesterol regulates autophagy in diverse ways across different models [[14], [15], [16], [17], [18]]. Therefore, we transfected BMSCs with GFP-mCherry-LC3 and examined them using confocal microscopy, which revealed a reduced number of autophagosomes in aged BMSCs (Fig. 1D). Concurrently, immunoblotting and immunofluorescence staining revealed reduced total autophagy and mitochondrial autophagy levels (Supplementary Fig. 2). Furthermore, we stimulated and depleted cholesterol levels in BMSCs and assessed autophagy and mitochondrial autophagy levels using western blotting (Fig. 1E). Cholesterol stimulation reduced LC3II levels, increased P62 accumulation, and decreased the expression of PINK1 and PARKIN, which are key markers of mitochondrial autophagy, indicating diminished total and mitochondrial autophagy. In contrast, cholesterol depletion via MβCD restored autophagy and mitochondrial autophagy levels. Transfection with GFP-mCherry-LC3 followed by confocal microscopy (Fig. 1F and G) revealed that cholesterol intervention reduced autophagosome formation, while MβCD-induced cholesterol depletion increased autophagosome formation, consistent with immunoblotting results. Moreover, cholesterol intervention reduced the colocalization of TOM20 with LC3 and the colocalization of mitochondria with lysosomes, whereas MβCD-induced cholesterol depletion increased their colocalization (Fig. 1F and G), reflecting cholesterol's influence on mitochondrial autophagy. Finally, we examined mitochondrial membrane potential (Supplementary Fig. 3A) and found that cholesterol levels similarly affected mitochondrial function. Based on these results, we concluded that elevated cholesterol levels in aged BMSCs inhibit autophagy.

Therefore, we administered the autophagy agonist rapamycin concurrently with cholesterol intervention to investigate whether autophagy suppression associated with elevated cholesterol levels was related to cholesterol-induced cellular senescence. β-galactosidase staining revealed that cholesterol intervention increased the proportion of SA-β-gal-positive cells, whereas rapamycin intervention reduced the number of these cells (Fig. 1H). Similarly, immunofluorescence staining for p21 and p53 (Fig. 1I and Supplementary Fig. 4A) revealed that rapamycin reversed cholesterol-induced increases in p21 and p53 expression. Furthermore, qRT-PCR analysis of IL-1β and IL-6 demonstrated that rapamycin suppressed the cholesterol-induced enhancement of SASP (Supplementary Fig. 3B). Chloroquine (CQ) is a specific downstream inhibitor of autophagy that is frequently used to study the effects of autophagy on cells [46]. MβCD treatment decreased the number of SA-β-gal positive cells in BMSCs, and the addition of CQ reversed the effect of MβCD intervention (Fig. 1H). However, the expression levels of p21 and p53 showed no statistically significant difference between the CQ-treated group and the combined CQ and MβCD group (Fig. 1I and Supplementary Fig. 4A), and qRT-PCR analysis of IL-1β and IL-6 exhibited a comparable trend. (Supplementary Fig. 3B). Given that cellular senescence in BMSCs compromises their osteogenic potential [2,3], we performed alkaline phosphatase (ALP) and alizarin red S (ARS) staining (Supplementary Fig. 4B). The results indicated that cholesterol stimulation reduced ALP and ARS levels in BMSCs, and this effect was reversed by rapamycin treatment. Similarly, MβCD intervention led to increased ALP and ARS levels, while CQ reversed this trend. Collectively, these findings indicated that elevated cholesterol levels in senescent BMSCs exacerbated cellular aging by inhibiting autophagy.

### 2.2 Cholesterol transporter ABCA1 suppresses autophagy by activating mTORC1 in aged BMSCs

**Fig. 2:**
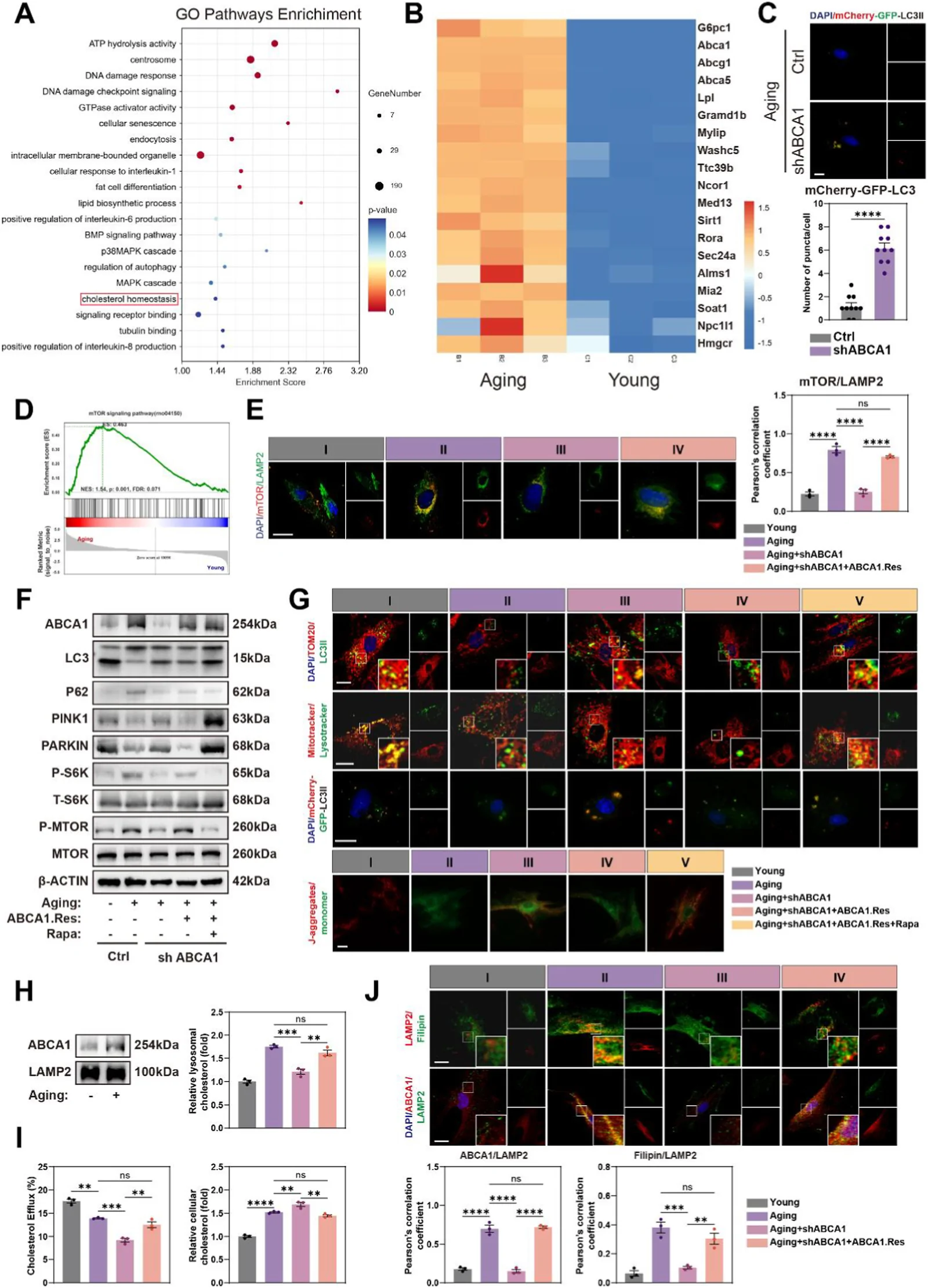
Cholesterol transporter ABCA1 suppresses autophagy by activating mTORC1 in aged BMSCs. **A.** GO Enrichment Analysis of BMSC Transcriptomics in Youth and Aging. **B.** Heat maps reveal gene expression profiles related to cholesterol homeostasis during aging. **C.** Representative confocal images of mCherry-GFP-LC3-labeled autophagosomes upon disruption of ABCA1 expression (scale bar = 10 μm) and semi-quantitative analysis (n = 11 biologically independent samples). **D.** Gene Set Enrichment Analysis (GSEA) of mTOR pathways. **E.** Representative confocal images of mTOR and LAMP2 staining following ABCA1 expression disruption and complementation (scale bar = 10 μm), along with colocalization analysis. **F.** Western blot analysis of mTORC1 activity and autophagy levels following disruption of ABCA1 expression and subsequent complementation. **G.** Representative confocal images of LC3II and TOM20 staining, representative confocal images of mCherry-GFP-LC3-labeled autophagosomes, representative confocal images of Lysotracker and Mitotracker, and representative fluorescence images of JC-1 mitochondrial membrane potential (scale bar = 10 μm), following ABCA1 expression disruption and complementation. **H.** Lysosomes were isolated from young and aged BMSCs to evaluate ABCA1 expression by Western blot analysis. Following ABCA1 silencing and rescue by ABCA1 overexpression, lysosomes were further isolated from BMSCs, and lysosomal cholesterol accumulation was quantified using an enzymatic assay (n = 3 independent biological replicates). **I.** After disrupting and complementing ABCA1 expression, measure total cellular cholesterol levels in BMSCs and determine cholesterol efflux rates (n = 3 biologically independent samples). **J.** Representative confocal images of ABCA1 staining and lysosomal staining alongside representative confocal images of Filipin staining and lysosomal staining (scale bar = 10 μm) following disruption and complementation of ABCA1 expression. Colocalization analysis of ABCA1 with lysosomal staining and Filipin with lysosomal staining (n = 3 independent biological samples). Data are presented as mean ± standard error of the mean (SEM). Statistical analysis was performed using two-tailed Student's t-test for comparisons between two groups and one-way analysis of variance (ANOVA) followed by Tukey's multiple comparisons test for comparisons among multiple groups. The number of biological replicates (n) is indicated in each figure legend. Statistical significance is indicated as *p < 0.05, **p < 0.01, and ***p < 0.001. BMSCs: Bone marrow-derived mesenchymal stem cells; Rapa: Rapamycin.

We performed transcriptomic analysis of young and aged BMSCs to identify the components of cholesterol metabolism that participate in autophagy inhibition and their effects. Compared to the young BMSCs, the aged BMSCs exhibited 2594 differentially up-regulated genes and 940 differentially downregulated genes (Supplementary Fig. 5A and B). Kyoto Encyclopedia of Genes and Genomes (KEGG) enrichment analysis revealed that cellular senescence and mTOR pathways were closely associated with BMSC aging (Supplementary Fig. 5C). Furthermore, consistent with previous studies in other models [8,47], Gene Ontology (GO) enrichment analysis indicated that cholesterol homeostasis contributes to the progression of BMSC aging (Fig. 2A). We then ranked the genes that were differentially expressed during cholesterol homeostasis (Fig. 2B), which revealed the elevated expression of the cholesterol transporters ABCA1, ABCG1, and ABCA5 in aged BMSCs. Subsequently, qRT-PCR analysis of these three proteins in aged BMSCs (Supplementary Fig. 6A) confirmed a significant upward trend in their expression. Additionally, the knockdown of these three proteins using short hairpin RNA revealed that only ABCA1 knockdown increased autophagosome formation (Fig. 2C and Supplementary Fig. 6B). Similarly, only ABCA1 knockdown enhanced mitochondrial autophagy and improved mitochondrial membrane potential (Supplementary Fig. 6C). Therefore, we proposed that ABCA1 participates in the inhibition of cholesterol-related autophagy.

KEGG enrichment analysis and GSEA of transcriptomics revealed that the mTOR pathway is involved in the senescence of BMSCs (Fig. 2D and Supplementary Fig. 5C). The mTORC1 complex is one of the primary regulators of anabolic and growth metabolism in the body [13], and functions as a nutrient sensor. When nutrients such as glucose, amino acids, or cholesterol are abundant, the mTORC1 complex is recruited to the lysosomal surface for activation, leading to autophagy inhibition and cellular senescence [48]. However, recent research has indicated that mTORC1 activation caused by excess nutrition shortens the lifespan of mice [49]. Therefore, we hypothesized that inhibition of autophagy and cellular senescence associated with cholesterol and cholesterol transporters may be linked to mTORC1 activation. Accordingly, we first examined mTOR colocalization with lysosomes (Fig. 2E). Interference with the expression of ABCA1 significantly reduced mTOR-lysosome colocalization, whereas ABCA1 overexpression restored this colocalization. Western blotting to detect mTORC1 activation and autophagy-related markers (Fig. 2F) revealed that aged BMSCs exhibited higher phosphorylation levels of s6k and mTOR compared to young BMSCs. Interference with ABCA1 expression reduced the phosphorylation levels, whereas rescuing ABCA1 expression reversed this trend. Similarly, interference with the expression of ABCA1 restored LC3II levels, reduced p62 accumulation, and increased PINK1 and PARKIN expression, indicating the recovery of both general autophagy and mitochondrial autophagy, a trend blocked by ABCA1 rescue overexpression. Furthermore, treatment with the mTORC1 inhibitor, rapamycin, reversed the suppression of autophagy induced by ABCA1 overexpression. Consistent with the immunoblotting results, we examined autophagosome formation and mitochondrial autophagy using immunofluorescence (Fig. 2G and Supplementary Fig. 7). These analyses revealed that interference with ABCA1 expression enhanced autophagy, while ABCA1 rescue overexpression conversely suppressed it. Moreover, rapamycin treatment relieved the ABCA1-induced inhibition. Collectively, these findings demonstrate that ABCA1 suppresses autophagy by activating mTORC1.

The accumulation of cholesterol in lysosomes can support mTORC1 activation and inhibit autophagy [11], and ABCA1 has been reported to act as a lysosomal cholesterol transporter in fibroblasts and astrocytes, supporting mTORC1 activation [7,8]. Therefore, we analyzed the abundance of ABCA1 in lysosomes via western blotting and found significantly elevated ABCA1 levels in the lysosomes of aged BMSCs (Fig. 2H). Filipin staining was performed to detect cholesterol and colocalization of ABCA1 with lysosomes (Fig. 2J). The results indicated that ABCA1 showed distinct colocalization with lysosomes in aged cells or that ABCA1 rescued overexpression, whereas cholesterol colocalization with lysosomes exhibited a trend consistent with ABCA1. Subsequently, we directly extracted lysosomes and measured their cholesterol levels (Fig. 2H), which correlated with the filipin staining results. Thus, we propose that ABCA1 may activate mTORC1 in senescent BMSCs by importing cholesterol into the lysosomes. The most well-known function of ABCA1 is cholesterol efflux across the plasma membrane [50]. Consequently, while the disruption of ABCA1 expression suppressed mTORC1 activation, it simultaneously reduced the cholesterol efflux rates in BMSCs and led to increased intracellular cholesterol levels (Fig. 2I).

### 2.3 ABCA1 localization is regulated by the palmitoylation/depalmitoylation cycle

**Fig. 3:**
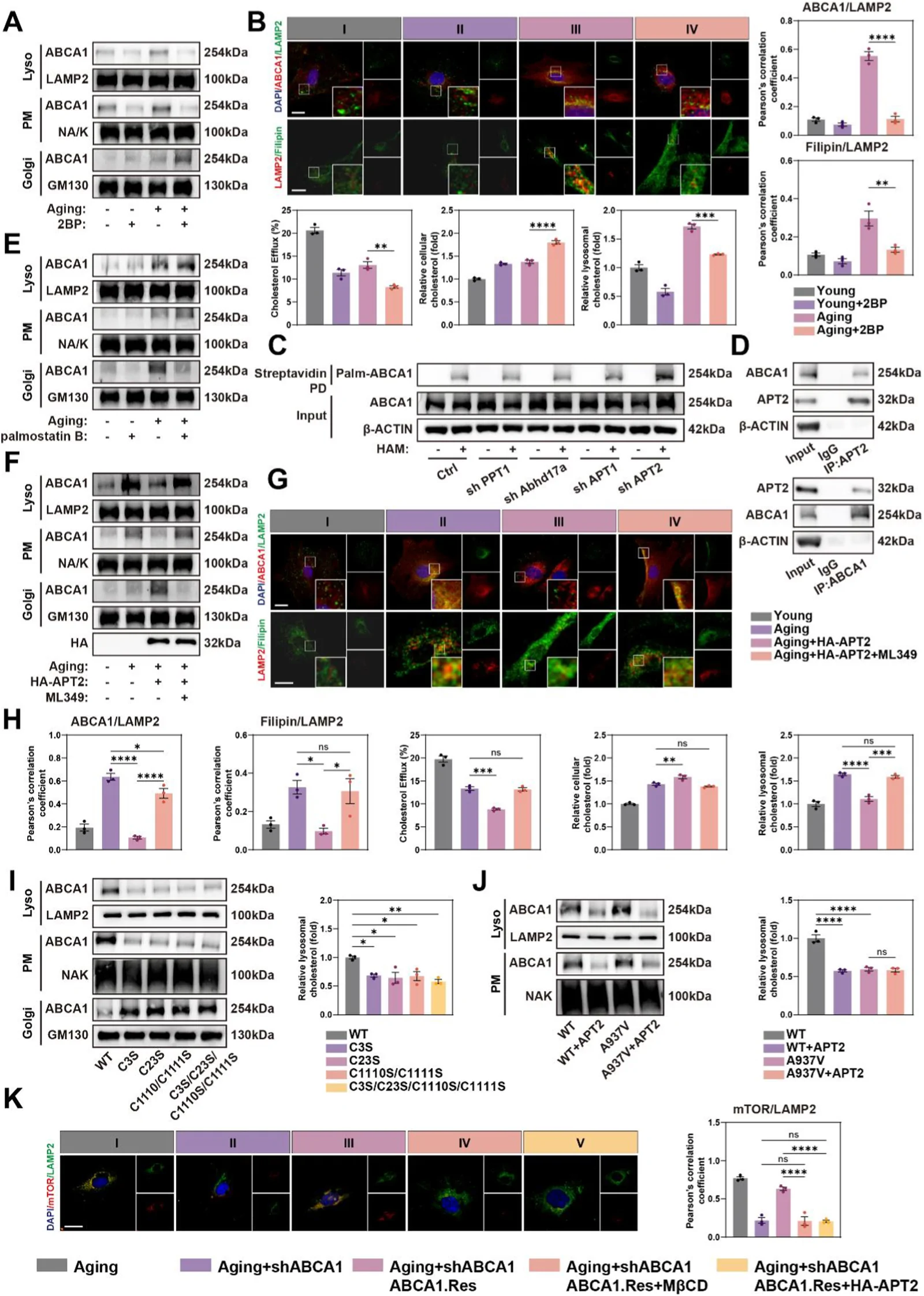
ABCA1 localization is regulated by a palmitoylation/depalmitoylation cycle, and its palmitoylation-dependent localization to lysosomes enables it to function as a cholesterol importer, activating mTORC1 and ultimately inhibiting autophagy. **A.** After blocking cellular palmitoylation with 2BP, ABCA1 levels in lysosomal, plasma membrane, and Golgi fractions were analyzed by Western blotting. **B.** After blocking cellular palmitoylation with 2-BP, representative confocal images showing ABCA1 distribution and Filipin-stained cholesterol in lysosomes, with quantitative analysis of their colocalization (scale bar = 10 μm) (n = 3 biologically independent samples). Cholesterol efflux capacity, cellular cholesterol levels, and lysosomal cholesterol accumulation were subsequently assessed (n = 3 biologically independent samples). **C.** ABE-mediated assessment of ABCA1 palmitoylation levels after interference with major depalmitoylation enzymes. **D.** Co-immunoprecipitation of ABCA1 and APT2. **E.** ABCA1 levels in lysosomal, plasma membrane, and Golgi fractions were analyzed by Western blotting after depalmitoylation inhibitor treatment. **F.** ABCA1 levels in lysosomal, plasma membrane, and Golgi fractions were analyzed by Western blotting following APT2 overexpression and treatment with an APT2 inhibitor. **G.** Representative confocal images of ABCA1 colocalization with lysosomal staining and Filipin colocalization with lysosomal staining after APT2 overexpression (scale bar = 10 μm). **H.** Cholesterol efflux capacity, cellular cholesterol levels, and lysosomal cholesterol accumulation were assessed following APT2 overexpression and treatment with an APT2 inhibitor (n = 3 biologically independent samples). **I.** Western blot analysis of the subcellular distribution of palmitoylation-deficient ABCA1 in lysosomal, plasma membrane, and Golgi fractions, together with the assessment of lysosomal cholesterol accumulation (n = 3 biologically independent samples). **J.** Western blot analysis of the subcellular distribution of transport-deficient mutant ABCA1 in lysosomal, plasma membrane, and Golgi fractions, together with the assessment of lysosomal cholesterol accumulation (n = 3 biologically independent samples). **K.** Representative confocal images (scale bar = 10 μm) and colocalization analysis of mTOR and LAMP2 staining following cholesterol depletion with MβCD or APT2 overexpression during ABCA1 rescue overexpression (n = 3 biologically independent samples). Data are presented as mean ± standard error of the mean (SEM). Statistical analysis was performed using two-tailed Student's t-test for comparisons between two groups and one-way analysis of variance (ANOVA) followed by Tukey's multiple comparisons test for comparisons among multiple groups. The number of biological replicates (n) is indicated in each figure legend. Statistical significance is indicated as *p < 0.05, **p < 0.01, and ***p < 0.001. BMSCs: Bone marrow-derived mesenchymal stem cells; 2BP: 2-Bromooctadecanoic acid; HAM: Hydroxylamine; PD: Pulldown; Palm-ABCA1: Palmitoylated ABCA1; MβCD represent cholesterol depletion, respectively; PM: Plasma membrane; Lyso: Lysosome; Golgi: Golgi apparatus.

Palmitoylation is a ubiquitous posttranslational modification that regulates protein function by influencing protein-membrane and membrane domain interactions, altering protein conformation, and affecting protein-protein interactions [31]. ABCA1 is a palmitoylated protein, and its palmitoylation is essential for its plasma membrane localization [34]. Based on previous studies, DHHC8 is the primary palmitoylating enzyme for ABCA1 in vivo [34]. Therefore, in this study, we employed the ABE method to detect palmitoylation of ABCA1 (Supplementary Fig. 8). The results indicate that palmitoylation occurs in ABCA1, and overexpression of DHHC8 enhances ABCA1 palmitoylation. Moreover treatment with the palmitoylation pharmacological inhibitor 2-bromopalmitic acid (2BP) blocked ABCA1 palmitoylation. ABCA1 enters endosomes and lysosomes via endocytosis through the plasma membrane [7]. We added 2BP or overexpressed ZDHHC8 to BMSCs, extracted the lysosomes, and performed immunoblotting to investigate the effect of palmitoylation on ABCA1 lysosomal localization (Fig. 3A and Supplementary Fig. 9). The results indicated that inhibiting palmitoylation significantly reduced lysosomal ABCA1, whereas enhancing palmitoylation increased lysosomal ABCA1 levels. Immunofluorescence staining was then performed to examine the effects of 2BP on lysosomal ABCA1 and cholesterol levels (Fig. 3B). Similar to the previous results, 2BP reduced lysosomal ABCA1 localization and lysosomal cholesterol levels, and direct measurement of lysosomal cholesterol levels showed a consistent trend (Fig. 3B). Additionally, we performed cell surface biotinylation and assessed the effects of 2BP and DHHC8 on ABCA1 plasma membrane localization via immunoblotting (Fig. 3A and Supplementary Fig. 9). The results demonstrated that the pharmacological inhibition of palmitoylation suppressed ABCA1 plasma membrane localization, ultimately impairing cellular cholesterol efflux and leading to cholesterol accumulation (Fig. 3B), which is consistent with the findings of previous studies [34]. The Golgi apparatus serves as the primary site for palmitoylation in mammalian cells, with the majority of the DHHC family of palmitoyltransferases being localized to membrane organelles dominated by the Golgi apparatus [31]. We hypothesized that ABCA1 undergoes palmitoylation by DHHC8 within the Golgi apparatus, enabling its transport to the plasma membrane via vesicular trafficking. Therefore, we isolated the Golgi apparatus from the BMSCs and performed immunoblotting (Fig. 3A). The results demonstrated that the pharmacological inhibition of palmitoylation increased ABCA1 expression in the Golgi apparatus, indicating that ABCA1 accumulated in the Golgi apparatus following palmitoylation blockade, which is consistent with our hypothesis.

Furthermore, we investigated the effect of depalmitoylation on the organellar distribution of ABCA1. First, we interfered with the expression of major depalmitoylation enzymes in vivo using short hairpin RNA and detected changes in ABCA1 palmitoylation levels using the ABE assay (Fig. 3C). Interference with acyl-protein thioesterase 2 (APT2) maximally increased ABCA1 palmitoylation. Additionally, co-immunoprecipitation (Fig. 3D) demonstrated an interaction between APT2 and ABCA1. These results indicate that APT2 is the primary depalmitoylase of ABCA1 in vivo. Next, we used immunoblotting to examine the effect of depalmitoylation on lysosomal ABCA1 levels (Fig. 3E, F and Supplementary Fig. 10). The potent depalmitoylation inhibitor palmostain B increased lysosomal localization of ABCA1, whereas APT2 overexpression decreased its localization. Immunofluorescence analysis of lysosomal ABCA1 and cholesterol further demonstrated that depalmitoylation contributes to reduced lysosomal ABCA1, thereby decreasing lysosomal cholesterol (Fig. 3G and H). Additionally, yhe direct measurement of lysosomal cholesterol levels confirmed this effect (Fig. 3H). Correspondingly, blocking de-palmitoylation increased plasma membrane ABCA1 and enhanced cholesterol efflux, whereas APT2 overexpression reduced plasma membrane ABCA1 and caused cellular cholesterol accumulation (Fig. 3E, F, and Supplementary Fig. 10). These results indicate that APT2-mediated depalmitoylation releases ABCA1 from the plasma membrane, endosomes, and lysosomes. We modulated depalmitoylation using the broad-spectrum depalmitoylation inhibitor Palmostatin B, APT2 overexpression or knockdown, and the APT2-specific inhibitor ML349, and subsequently examined the intracellular distribution of ABCA1 by Western blotting (Fig. 3E, F and Supplementary Fig. 10). Notably, enhanced depalmitoylation increased Golgi-associated ABCA1 expression, whereas inhibition of depalmitoylation reduced it. We hypothesized that this may represent a palmitoylation/depalmitoylation cycle for ABCA1, whereby ABCA1 from the plasma membrane and endosomes/lysosomes undergoes depalmitoylation, returns to the Golgi apparatus for repalmitoylation, and subsequently relocalizes to the plasma membrane.

To further validate our findings, we employed CSS-Palm 4.0 to predict potential palmitoylation sites in ABCA1(Supplementary Fig. 11A). The predicted high-scoring sites were consistent with previously reported palmitoylation sites [34]. These candidate residues were subsequently mutated to serine to disrupt their palmitoylation. Acyl-biotin exchange (ABE) assays were then performed to assess changes in ABCA1 palmitoylation levels (Supplementary Fig. 11B). Consistent with previous reports, mutation of the three major palmitoylation sites significantly reduced ABCA1 palmitoylation, whereas simultaneous mutation of all three sites almost completely abolished ABCA1 palmitoylation. We next investigated the effects of these palmitoylation site mutations on the subcellular distribution of ABCA1. Mutation of these sites impaired ABCA1 localization to lysosomal and plasma membrane compartments, reduced lysosomal cholesterol accumulation, and ultimately resulted in ABCA1 retention in the Golgi apparatus, consistent with our previous findings (Fig. 3I). Furthermore, mutation of the ABCA1 palmitoylation sites also reduced mTORC1 activation, indicating that ABCA1 palmitoylation is required for maintaining mTORC1 signaling activity (Supplementary Fig. 11C).

To determine whether ABCA1 functions as a lysosomal cholesterol importer, we generated a transport-deficient ABCA1 mutant with impaired cholesterol transport activity. Western blot analysis showed that this mutant exhibited no apparent changes in subcellular distribution and retained responsiveness to APT2 overexpression. However, loss of ABCA1 transport activity markedly reduced lysosomal cholesterol accumulation in BMSCs. Moreover, disruption of ABCA1 transport function abolished the effect of the depalmitoylating enzyme APT2 on lysosomal cholesterol levels, indicating that ABCA1 mediates lysosomal cholesterol import in a palmitoylation-dependent manner (Fig. 3J).

Finally, while rescuing ABCA1 expression, we added MβCD or overexpressed APT2 separately. MβCD depleted intracellular cholesterol, whereas APT2 reduced ABCA1 localization in the endomembrane system, including lysosomes. Subsequently, we observed the co-localization of mTOR and lysosomes using confocal microscopy (Fig. 3K). MβCD intervention and APT2 overexpression eliminated the enhanced mTOR-lysosome colocalization induced by ABCA1 rescue overexpression. Similar findings were observed in autophagosomes by confocal microscopy (Supplementary Fig. 12). Collectively, these results indicate that ABCA1-mediated autophagy inhibition depends on its lysosomal localization and cellular cholesterol levels.

**Scheme 2:**
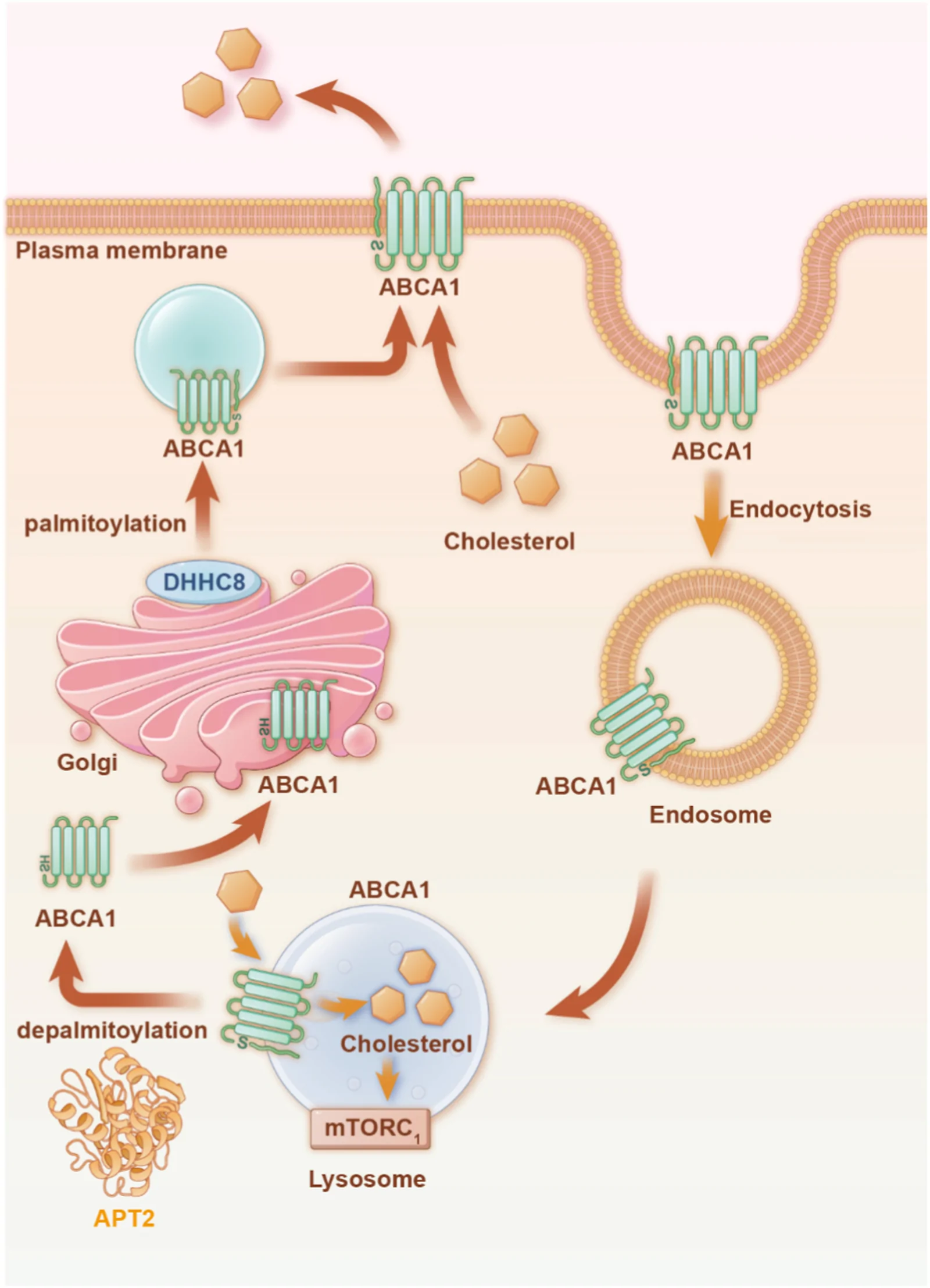
Schematic model illustrating how ABCA1 palmitoylation cycling regulates its trafficking among intracellular organelles.

The transport, sorting, and degradation of ABCA1 constitutes a highly dynamic and complex system. Our findings indicate that the palmitoylation/depalmitoylation cycle governs the interorganelle transport of ABCA1. Following endocytosis, ABCA1 is transported to the endolysosomal membrane [7]. Both plasma membrane and endolysosomal ABCA1 are subsequently depalmitoylated by APT2, reducing their membrane association and facilitating their recycling to the Golgi apparatus, where they may undergo another round of palmitoylation. When ABCA1 resides in the plasma membrane, it performs its classical function of cholesterol efflux. Upon entering the endosome via endocytosis and subsequently reaching the lysosomal surface, it acts as a coactivator of mTORC1 signaling. Therefore, we propose that controlling the organellar distribution of ABCA1 through palmitoylation regulation is a feasible strategy for alleviating mTORC1-mediated autophagy inhibition while preserving plasma membrane ABCA1-mediated cholesterol efflux (Scheme 2).

### 2.4 Engineered organelle-targeted depalmitoylation enzymes redirect ABCA1 from lysosomes to the plasma membrane

**Fig. 4:**
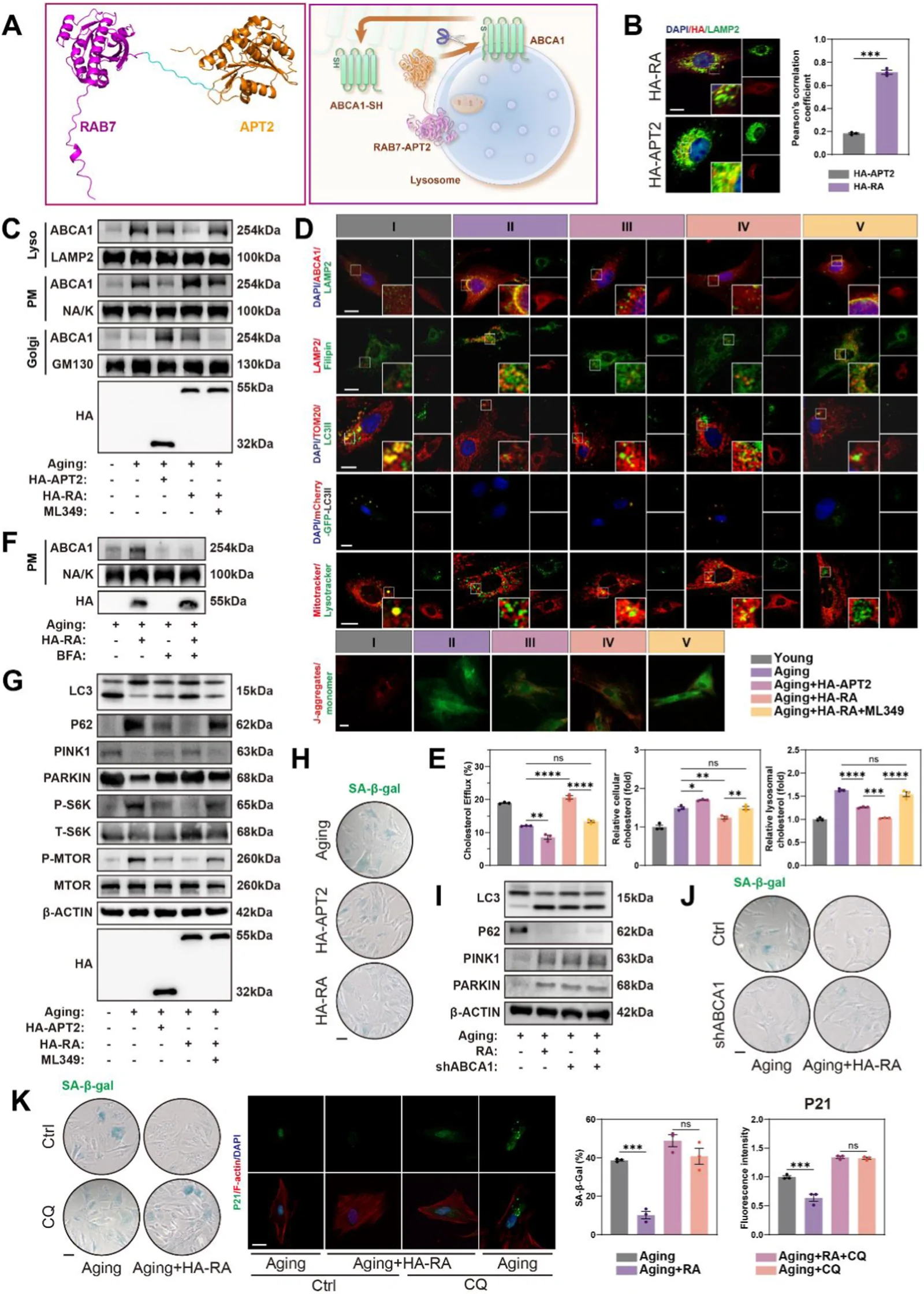
Engineered organelle-targeted depalmitoylation enzymes redirect ABCA1 from lysosomes to the plasma membrane, enhancing cholesterol efflux while improving cellular senescence through restored autophagy. **A.** Schematic illustration depicting the structural features of RAB7-APT2 and the proposed mechanism by which RAB7 recruits APT2 to selectively mediate depalmitoylation of lysosome-localized ABCA1. **B.** Representative confocal images of APT2 staining with LAMP2 and RA staining with LAMP2 (scale bar = 10 μm), with colocalization analysis performed using ImageJ (n = 3 biologically independent samples). **C.** Western blot analysis of the subcellular distribution of ABCA1 among lysosomal, plasma membrane, and Golgi fractions following APT2 or RAB7–APT2 (RA) overexpression. **D.** Representative confocal microscopy images showing ABCA1/lysosome and Filipin/lysosome colocalization, LC3/mitochondria colocalization, lysosome–mitochondria interactions, and autophagic flux assessment (scale bar = 10 μm). **E.** Assessment of cholesterol efflux capacity, cellular cholesterol levels, and lysosomal cholesterol accumulation in BMSCs following RA or APT2 overexpression (n = 3 biologically independent samples). **F.** Western blot analysis demonstrating that brefeldin A treatment abolished the RA-mediated increase in plasma membrane-localized ABCA1. **G.** Western blot analysis of the effects of RA or APT2 overexpression on mTORC1 activity and autophagy-related protein expression in aged BMSCs. **H.** Representative SA-β-gal staining images of BMSCs following overexpression of RA or APT2. **I.** Western blot analysis of autophagy-related protein expression in BMSCs following RA overexpression with or without ABCA1 knockdown. **J.** Representative images of SA-β-gal staining in BMSCs following RA overexpression with ABCA1 knockdown. **K.** Representative images of SA-β-gal staining and p21 immunofluorescence staining (scale bar = 10 μm), together with their semi-quantitative analyses (n = 3 biologically independent samples), in BMSCs following RA overexpression in the presence of the autophagy inhibitor chloroquine. Data are presented as mean ± standard error of the mean (SEM). Statistical analysis was performed using two-tailed Student's t-test for comparisons between two groups and one-way analysis of variance (ANOVA) followed by Tukey's multiple comparisons test for comparisons among multiple groups. The number of biological replicates (n) is indicated in each figure legend. Statistical significance is indicated as *p < 0.05, **p < 0.01, and ***p < 0.001. BMSCs: Bone marrow-derived mesenchymal stem cells; HA-RA and HA-APT2: HA-tagged RAB7-APT2 engineered enzyme and APT2, respectively; PM: Plasma membrane; Lyso: Lysosome; BFA: Brefeldin A; SA-β-Gal: Senescence-associated β-galactosidase; CQ: Chloroquine.

Research has already attempted to regulate disease progression by altering protein palmitoylation levels. One study inhibited melanoma progression by delivering the palmitoylation inhibitor 2BP to suppress PD-L1 membrane localization and exosomal targeting [37], Similarly, increasing brain palmitoylation improved behavioral and neuropathological outcomes in mice with Huntington's disease mice [51]. However, in the case of ABCA1, direct pharmacological inhibition of palmitoylation or overexpression of a palmitoylation-inhibiting enzyme reduces both lysosomal and plasma membrane localization of ABCA1, impairing the cellular cholesterol efflux capacity. Numerous studies indicate that elevated intracellular cholesterol levels weaken osteogenic differentiation while enhancing adipogenic differentiation in bone marrow mesenchymal stem cells (BMSCs) [[28], [29], [30]]. Therefore, we recognize the need for more precise interventions that target the ABCA1 cycle. Previous studies have demonstrated that attaching specific sequences or nanobodies to depalmitoylation enzymes enables a more controlled and precise regulation of depalmitoylation levels [52,53]. Therefore, we engineered a depalmitoylating enzyme, RAB7-APT2 (RA), by flexibly connecting the RAB7 sequence to the carboxyl-terminus of APT2. We aimed to preferentially direct RA to late endosomes and lysosomes via the RAB7 sequence, thereby reducing the palmitoylation levels of lysosomal ABCA1 without significantly affecting plasma membrane ABCA1 (Fig. 4A). Immunofluorescence analysis of RAB7-linked RA co-localization with lysosomes (Fig. 4B) revealed significantly higher lysosomal colocalization compared to APT2. We further assessed the overall palmitoylation of ABCA1 by ABE (Supplementary Fig. 13). Compared with APT2, milder RA-mediated ABCA1 depalmitoylation is likely due to its organelle-specific depalmitoylation effect. Furthermore, we examined the organellar localization of ABCA1 using immunoblotting (Fig. 4C). Compared to APT2, RA reduced lysosomal ABCA1. Contrary to APT2, RA did not decrease plasma membrane ABCA1 but instead increased it relative to the aging-only group. Similar to APT2 treatment, RA treatment increased ABCA1 levels in the Golgi apparatus. We propose that these findings indicate that RA releases lysosomal ABCA1, potentially allowing its repalmitoylation upon return to the Golgi apparatus and subsequent re-localization to the plasma membrane. The immunofluorescence and enzymatic assays yielded comparable results (Fig. 4D, E and Supplementary Fig. 14A); RA effectively reduced the co-localization of ABCA1 with lysosomes and decreased lysosomal cholesterol. To exclude the potential contribution of RAB7 itself, we further examined the effects of RAB7 overexpression alone and found that it neither significantly altered the subcellular distribution of ABCA1 nor affected autophagy levels in aged BMSCs (Supplementary Fig. 14B and C). Consistent with the increased ABCA1 plasma membrane localization, RA intervention, in contrast to APT2, increased the cholesterol efflux rate and reduced intracellular cholesterol in BMSCs (Fig. 4E). Brefeldin A (BFA), a lactone antibiotic that blocks the Golgi secretory function, is frequently used to study protein transport [54]. To investigate whether the RA-mediated increase in plasma membrane ABCA1 resulted from the re-palmitoylation of lysosomal ABCA1 in the Golgi apparatus, we blocked Golgi transport using BFA. Immunoblotting revealed no significant difference between the aging and RA-treated groups after BFA intervention (Fig. 4F). This indicates that the RA-mediated increase in ABCA1 plasma membrane localization depends on the secretory function of the Golgi apparatus. Therefore, we proposed that lysosomal ABCA1 is released by RA and transported to the plasma membrane via re-palmitoylation in the Golgi apparatus.

Furthermore, we examined mTORC1 activity and autophagy levels following RA intervention using Western blot analysis (Fig. 4G). The results demonstrated that RA inhibited mTORC1 activity more potently than APT2 and effectively restored both total and mitochondrial autophagy. To exclude the possibility that LC3-II accumulation resulted from impaired autophagic flux, we analyzed autophagy-related protein expression and autophagic flux at early (4 h) and late (24 h) time points following RA treatment. The results confirmed that RA enhanced autophagic activity rather than causing autophagosome accumulation due to blocked degradation (Supplementary Fig. 18). Immunofluorescence analysis also demonstrated that RA intervention induced more autophagosome formation, increased mitochondrial-LC3 and lysosomal colocalization to a greater extent, and better restored the mitochondrial membrane potential compared to APT2 (Fig. 4D and Supplementary Fig. 14). We also examined the effects of RA on cellular senescence. Immunofluorescence analysis revealed that RA significantly reduced the fluorescence intensity of P21 and P53 (Supplementary Fig. 15B). Similarly, RA decreased the percentage of SA-β-gal-positive cells (Fig. 4H and Supplementary Fig. 15A) and reduced the expression of SASP inflammatory factors (Supplementary Fig. 15C). To determine whether the therapeutic effects of RA are dependent on ABCA1, we performed ABCA1 knockdown using shABCA1 during RA treatment. Notably, ABCA1 knockdown abolished the differences in autophagy levels and cellular senescence between RA-treated and untreated groups, indicating that the therapeutic effects of RA are mediated through its regulation of ABCA1 (Fig. 4I, J and Supplementary Fig. 16). We next investigated the causal relationship between RA-induced autophagy activation and cellular senescence by pharmacologically inhibiting autophagy with chloroquine (CQ). CQ abolished the RA-mediated reduction in P21 and p53 fluorescence intensities (Fig. 4K and Supplementary Fig. 17A), reversed the improvement in SA-β-gal positivity following RA intervention (Fig. 4K) and restored SASP to elevated levels (Supplementary Fig. 17B). We further validated our findings using primary BMSCs isolated from young (4-week-old) and aged (12-month-old) rats. Consistent with our previous results, RA promoted ABCA1 redistribution, reduced lysosomal cholesterol accumulation, and restored autophagic activity in aged BMSCs (Supplementary Fig. 19). Taken together, we propose that the engineered palmitoylase, RA, modulates the respective proportions of the ABCA1 pool in the plasma membrane and lysosomes through organelle-targeted depalmitoylation of senescent BMSCs. This shifts ABCA1 recycling toward the plasma membrane, enhances cholesterol efflux, and improves cellular senescence by restoring autophagy.

### 2.5 Synthesis and characterization of LNP@A loaded with RA mRNA

**Fig. 5:**
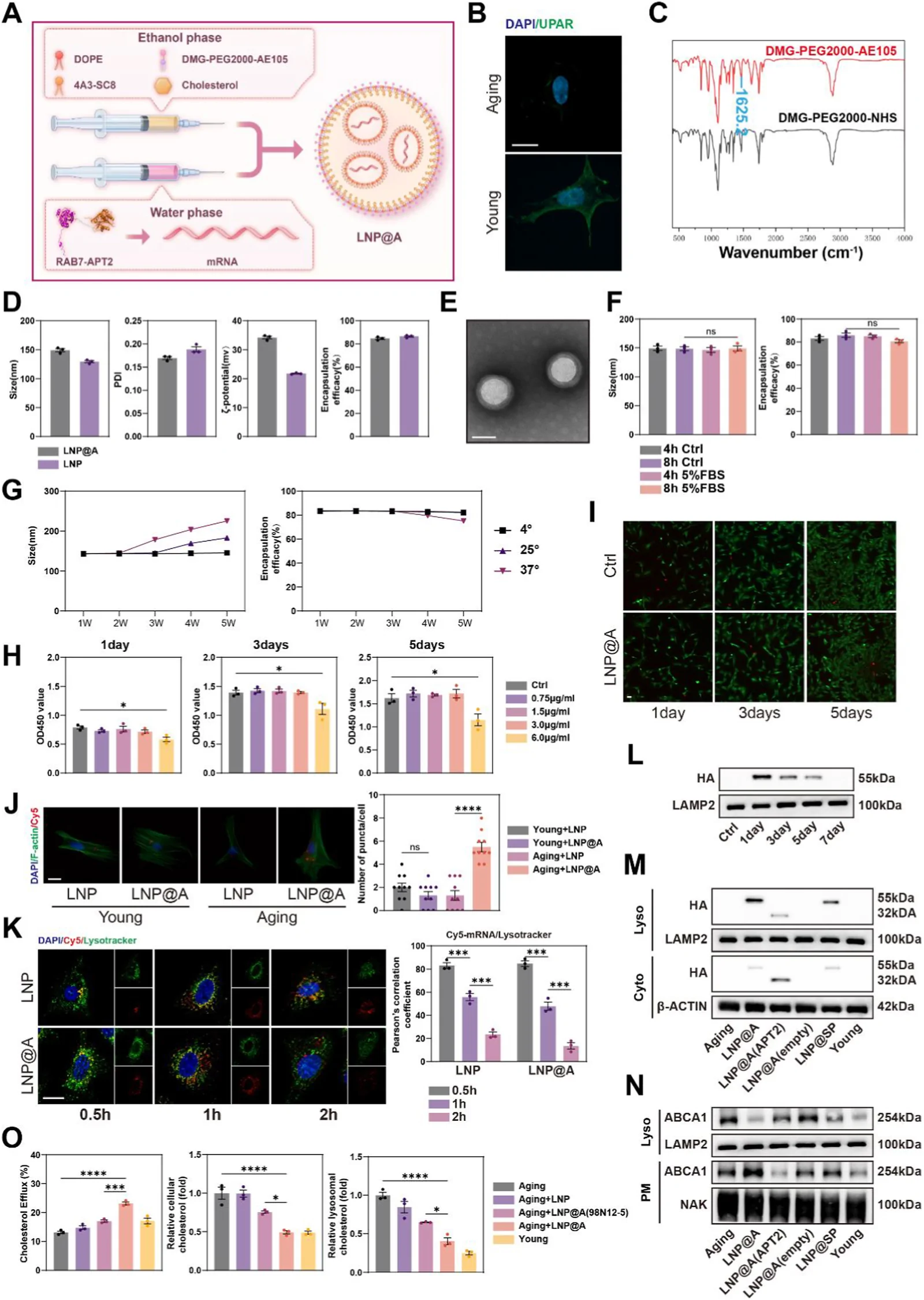
Synthesis and Characterization of LNP@A Loaded with RA mRNA. **A.** Components of LNP@A and schematic diagram of synthesis. **B.** Representative uPAR immunofluorescence staining images of young or aged BMSCs (scale bar = 10 μm) and semi-quantitative analysis (n = 3 biologically independent samples). **C.** Infrared spectroscopy analysis of DMG-PEG2000-AE105 before and after synthesis. **D.** Particle size, PDI, zeta potential, and encapsulation efficiency of LNP@A. **E.** Representative TEM image of LNP@A (scale bar = 100 nm). **F.** Assessment of the serum stability of LNP@A by measuring particle size and encapsulation efficiency in 5% FBS (n = 3). **G.** Evaluation of the storage stability of LNP@A (n = 3). **H.** BMSC viability assessed by CCK-8 assay after 1, 3, and 5 days of LNP@A treatment at different concentrations (n = 3 biologically independent samples). **I.** Representative fluorescence images of live cells (green) stained with calcein and dead cells (red) stained with propidium iodide after LNP@A treatment at 3 μg/ml (scale bar = 20 μm). **J.** Representative fluorescence images of BMSCs internalizing fluorescent liposomes after 24-h incubation (scale bar = 10 μm) and quantitative analysis (n = 3 independent biological replicates). **K.** Representative confocal microscopy images of LNP@A endosomal escape (scale bar = 20 μm) and the corresponding semi-quantitative analysis (n = 3 biologically independent samples). **L.** Time-dependent analysis of protein expression following LNP@A-mediated mRNA delivery by Western blotting. **M.** Western blot analysis of the subcellular distribution of RA in lysosomal and cytoplasmic fractions following LNP@A treatment. Aged BMSCs were used in all groups except the young control group. **N.** Western blot analysis of the changes in ABCA1 distribution between lysosomal and plasma membrane fractions following LNP@A treatment. Aged BMSCs were used in all groups except the young control group. **O.** Assessment of cholesterol efflux capacity, cellular cholesterol levels, and lysosomal cholesterol accumulation in BMSCs after LNP@A treatment (n = 3 independent biological replicates). Data are presented as mean ± standard error of the mean (SEM). Statistical analysis was performed using two-tailed Student's t-test for comparisons between two groups and one-way analysis of variance (ANOVA) followed by Tukey's multiple comparisons test for comparisons among multiple groups. The number of biological replicates (n) is indicated in each figure legend. Statistical significance is indicated as *p < 0.05, **p < 0.01, and ***p < 0.001. BMSCs: Bone marrow-derived mesenchymal stem cells.

We believe that expressing RA may be a viable strategy for improving age-related bone defects. Therefore, we sought to develop an in vivo mRNA delivery system for RA to redirect ABCA1 trafficking. Compared to delivering DNA, delivering mRNA does not require crossing the nuclear membrane and can function within the cytoplasm [55]; thus, the primary challenge in delivering mRNA is to efficiently traverse the plasma membrane at a minimal cost. Liposomes are artificial vesicles with a lipid bilayer membrane that offer advantages such as transient expression and safe nucleic acid delivery [55,56]. Therefore, we designed targeted delivery liposomes carrying mRNA for in vivo expression. Linking targeting peptides to the LNP surface enhances their targeting ability and improves uptake by target tissues or cells [57,58]. Previous studies have shown that the urokinase-type plasminogen activator receptor (uPAR) is highly expressed in senescent cells [59,60], and the modified uPAR-targeting peptide AE-105 enables the in vivo imaging of uPAR-associated tissues [61,62]. We observed elevated uPAR expression on the surface of aged BMSCs using immunofluorescence (Fig. 5B). Subsequently, DMG-PEG2000-NHS was covalently conjugated to AE-105 using EDC chemistry. Fourier transform infrared spectroscopy analysis revealed a characteristic amide bond peak at 1625.2 cm^−1^ (Fig. 5C), confirming the successful synthesis of DMG-PEG2000-AE105. Following a previous method [63,64], we dissolved 4A3-SC8, DOPE, and cholesterol, and synthesized DMG-PEG2000-AE105 in ethanol as the ethanol phase at a molar ratio of 50/10/38.5/1.5. The RA mRNA was dissolved in citrate buffer (pH4) as an aqueous phase. The flow rate ratio of the aqueous phase to ethanol phase was 3:1. and the mRNA-to-total lipid mass ratio was 1:40. The aqueous and ethanol phases were mixed using microfluidic technology, followed by dialysis to obtain the RA-loaded mRNA-targeted liposomes LNP@A (Fig. 5A). Nucleic acid gel electrophoresis revealed bands only in the mRNA and LNP@A groups, confirming successful mRNA loading (Supplementary Fig. 21). We characterized the particle size, PDI, and zeta potential of LNP@A using dynamic light scattering (DLS) (Fig. 5D). LNP@A exhibited an average particle size of approximately 140 nm, PDI < 0.3, and zeta potential > 30mv, indicating a reasonable size, reliable uniformity, and stability. Transmission electron microscopy (TEM) further characterized the size and morphology of LNP@A (Fig. 5E).

To evaluate the serum stability of LNP@A, we measured the particle size and encapsulation efficiency of LNP@An under 5% FBS conditions. The results demonstrated that LNP@A maintained stable physicochemical properties in the presence of serum, indicating good serum stability (Fig. 5F). In addition, we assessed the storage stability of LNP@A at 4°C, 25°C, and 37°C. LNP@A exhibited long-term stability when stored at 4°C, suggesting its potential for practical storage and application (Fig. 5G). Moreover, LNP@A prepared from different batches exhibited excellent batch-to-batch consistency, with comparable particle size, polydispersity index (PDI), zeta potential, and encapsulation efficiency, demonstrating the reproducibility and reliability of the LNP@A formulation (Supplementary Fig. 22).

To assess the biocompatibility of LNP@A, we evaluated its cellular effects using a CCK8 assay (Fig. 5H). The results indicated that LNP@A had no significant effect on cell viability at concentrations below 6.0 μg/ml. Following BMSC treatment with 3.0 μg/ml LNP@A, cell death staining was performed (Fig. 5I and Supplementary Fig. 23A). Consistent with CCK8 results, 3.0 μg/ml LNP@A showed no significant effect on cell death. LNP have been reported to induce the production of inflammatory cytokines [[64], [65], [66], [67], [68]]; however, the ionizable lipid 4A3-SC8 has been reported to mediate mild endosomal escape by forming smaller pores in the endosomal membrane, thereby eliciting minimal inflammatory responses [64]. This is one of the reasons for the selected 4A3-SC8. We employed multiple ionizable lipids (98N12-5, C12-200, cKK-E12) to construct empty LNPs and examined IL-1β and IL-6 expression (Supplementary Fig. 23B). The results indicated that LNP@A formulated with 4A3-SC8 had no significant effect on cytokine production, whereas LNPs using other lipids induced higher cytokine expression.

We conjugated Cy5 to DOPE and prepared liposomes with and without AE105 attachment. Endocytosis was assessed by fluorescence microscopy (Fig. 5J). The results demonstrated that only LNP@A containing AE105 exhibited robust endocytosis in aged BMSCs, whereas LNP without AE105 or in young BMSCs showed minimal internalization. We further evaluated the cellular uptake of LNP@A and scrambled peptide-modified LNP (LNP@SP). Consistently, LNP@A exhibited enhanced uptake by uPAR-expressing senescent BMSCs, confirming the specific targeting capability of the LNP platform toward senescent cells (Supplementary Fig. 24). Furthermore, we evaluated the endosomal escape capability of LNP@A. The results demonstrated that LNP@A exhibited efficient endosomal escape properties, regardless of the presence or absence of the AE105 targeting peptide (Fig. 5K). We further characterized the expression kinetics of RA mediated by LNP@A in BMSCs. RA expression remained relatively high during the first three days following LNP@A treatment and was completely diminished by day 7, demonstrating a transient and controllable expression profile. This transient expression pattern may reduce potential risks associated with prolonged expression of exogenous proteins (Fig. 5L).

To further determine the subcellular distribution of RA expressed by the LNP@A system, we used LNP@A carrying APT2 mRNA as a control and analyzed the intracellular localization of RA following LNP@A treatment. Western blot analysis of organelle fractions revealed that APT2 was predominantly localized in the cytoplasm, with only a small proportion detected in lysosomes. In contrast, the majority of RA was enriched in lysosomes, whereas only a minor fraction was present in the cytoplasm. These results demonstrate the effectiveness of our RAB7-mediated protein organelle-targeting strategy (Fig. 5M).

Finally, we examined the effect of LNP@A loaded with RA-mRNA on ABCA1 localization (Fig. 5N and Supplementary Fig. 25). Compared with all control groups, LNP@A exhibited the strongest ability to promote ABCA1 redistribution from lysosomes to the plasma membrane. Consistently, LNP@A maximally reduced lysosomal cholesterol levels, enhanced cholesterol efflux in senescent BMSCs, and significantly decreased intracellular cholesterol levels (Fig. 5O). Collectively, these results demonstrate that LNP@A successfully promotes the relocalization of ABCA1 to the plasma membrane. Therefore, we propose that LNP@A is a viable strategy for improving cellular senescence in aged BMSCs and enhancing their osteogenic potential.

### 2.6 LNP@A restore the osteogenic potential aged BMSCs in vitro

**Fig. 6:**
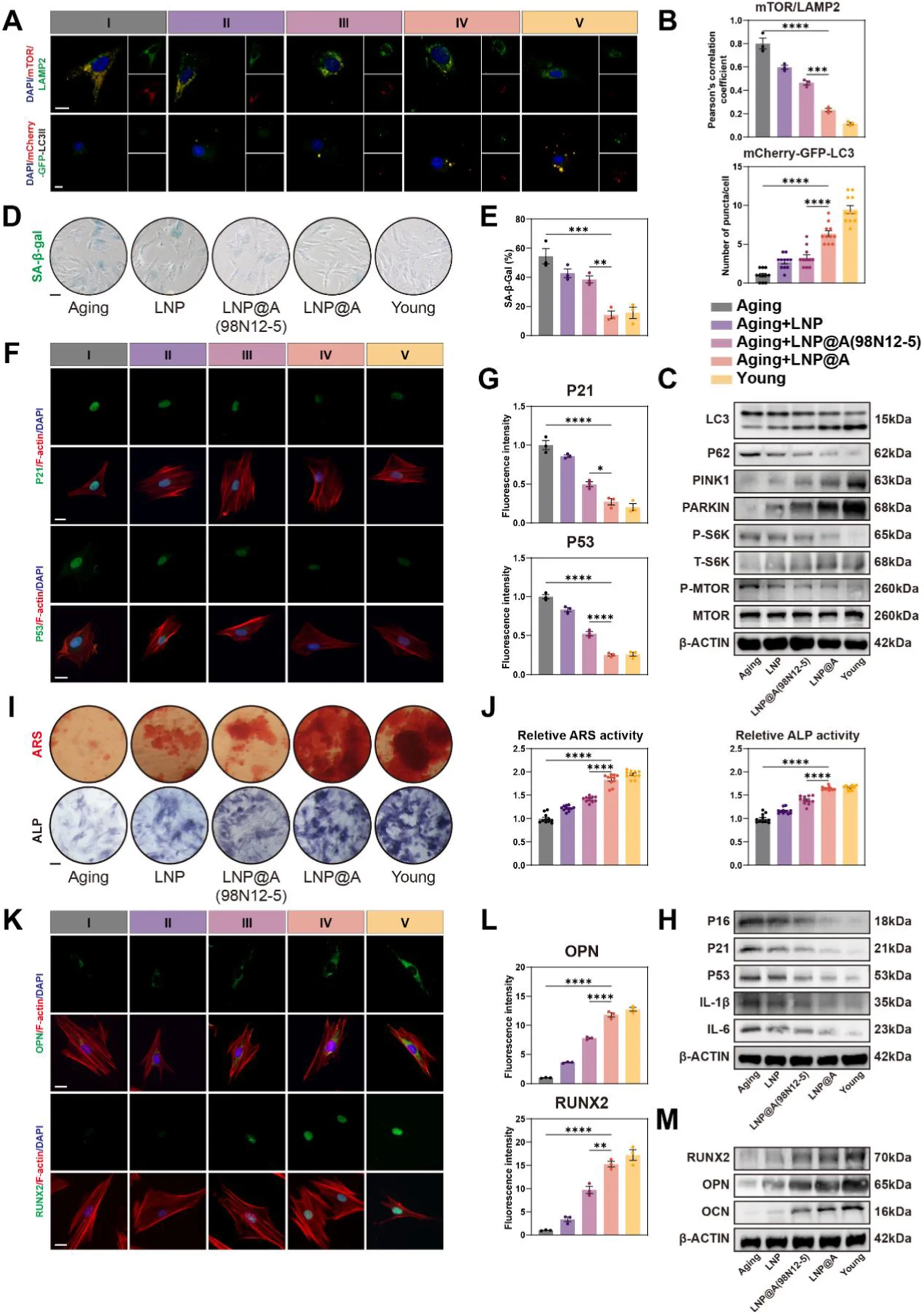
LNP@A restores autophagy in aged BMSCs in vitro, alleviates cellular senescence, and reactivates the osteogenic potential of aged BMSCs. **A.** Representative confocal images of mTOR and LAMP2 staining, and mCherry-GFP-LC3-labeled autophagosomes following treatment with untargeted LNP, LNP@A with 98N12-5, and LNP@A (scale bar = 10 μm). **B.** Colocalization analysis of mTOR and LAMP2 staining (n = 3 biologically independent samples), and semi-quantitative analysis of mCherry-GFP-LC3-labeled autophagosomes (n = 11 biologically independent samples). **C.** Western blot analysis of mTORC1 activity and autophagy levels following intervention with LNP without targeting peptide, LNP@A with 98N12-5, and LNP@A. **D.** Representative images of SA-β-gal staining following intervention with LNP without targeting peptide, LNP@A with 98N12-5, and LNP@A (scale bar = 20 μm). **E.** Quantitative analysis of SA-β-gal (n = 3 independent biological samples). **F.** Representative immunofluorescence images of P21 and p53 staining after treatment with untargeted LNP, 98N12-5 LNP@A, and LNP@A (scale bar = 10 μm). **G.** Semi-quantitative analysis of P21 and P53 staining (n = 3 independent biological replicates). **H.** Western blot analysis of senescence and SASP inflammatory factors following intervention with untargeted LNP, 98N12-5 LNP@A, and LNP@A. **I.** Representative images of ARS and ALP staining after intervention with untargeted LNP, 98N12-5 LNP@A, and LNP@A (scale bar = 20 μm). **J.** Quantitative analysis of ALP and ARS (n = 11 biologically independent samples). **K.** Representative immunofluorescence images of OPN and RUNX2 staining after intervention with LNP without targeting peptide, LNP@A with 98N12-5, and LNP@A (scale bar = 10 μm). **L.** Semi-quantitative analysis of OPN and RUNX2 staining (n = 3 biologically independent samples). **M.** Western blot analysis of osteogenic markers following intervention with untargeted LNP, 98N12-5 LNP@A, and LNP@A. Data are presented as mean ± standard error of the mean (SEM). Statistical analysis was performed using two-tailed Student's t-test for comparisons between two groups and one-way analysis of variance (ANOVA) followed by Tukey's multiple comparisons test for comparisons among multiple groups. The number of biological replicates (n) is indicated in each figure legend. Statistical significance is indicated as *p < 0.05, **p < 0.01, and ***p < 0.001. BMSCs: Bone marrow-derived mesenchymal stem cells; SA-β-Gal: Senescence-associated β-galactosidase; ALP: Alkaline phosphatase; ARS: Alizarin red S.

First, we performed western blotting to detect mTORC1 activation and autophagy-related markers in aged BMSCs after LNP@A treatment to validate the efficacy of LNP@A in aged BMSCs (Fig. 6C and Supplementary Fig. 26A). The results showed that LNP@A maximally reduced the phosphorylation levels of s6k and mTOR, indicating decreased mTORC1 activity. Furthermore, LNP@A significantly increased LC3II levels, elevated PINK1 and PARKIN expression, and reduced P62 accumulation compared to the control group, demonstrating enhanced total autophagy and mitophagy in senescent BMSCs. Immunofluorescence analysis of mTOR-lysosome colocalization (Fig. 6A and B) similarly demonstrated that LNP@A maximally reduced mTOR activation in lysosomes, and maximally increased autophagosome formation, rather than other controls (Fig. 6A and B). Co-localization of mitochondria with LC3 and lysosomes exhibited a similar trend (Supplementary Fig. 26B and C), indicating that LNP@A maximally restored mitochondrial autophagy, ultimately improving mitochondrial membrane potential (Supplementary Fig. 26D). These results demonstrate that LNP@A effectively restores general and mitochondrial autophagy in aged BMSCs.

Previous work has demonstrated that RA improves cellular senescence in aged BMSCs by restoring autophagy (Fig. 4G–K); Therefore, we examined the effect of LNP@A intervention on cellular senescence in aged BMSCs. LNP@A significantly reduced the level of SA-β-gal positive cells (Fig. 6D and E). Similarly, the immunofluorescence analysis of p21 and p53 (Fig. 6F and G) revealed that LNP@A maximally reduced their expression levels. Finally, Western blot analysis of the senescence and SASP factors (Fig. 6H) demonstrated that LNP@A maximally decreased their expression. These results indicated that LNP@A effectively ameliorated cellular senescence in aged BMSCs.

Accumulation of senescent BMSCs often leads to diminished osteogenic potential [2,3]. Previous studies have demonstrated that restoring autophagy or reversing cellular senescence can enhance the osteogenic potential of senescent BMSCs [[69], [70], [71], [72]], therefore, we propose that LNP@A can improve the osteogenic potential of senescent BMSCs. We used alizarin red S (ARS) and alkaline phosphatase (ALP) staining to assess the effect of LNP@A on the osteogenic potential of aged BMSCs (Fig. 6I and J). The results indicated that the LNP@A-treated group exhibited stronger osteogenic capacity than the control group. The immunofluorescence analysis revealed that LNP@A increased the expression of osteogenesis-related proteins (Fig. 6K and L). We further assessed the osteogenic potential of aged BMSCs using western blotting. Similarly, the LNP@A-treated group showed the maximum increase in the expression of osteogenic proteins, including RUNX2, OPN, and OCN (Fig. 6M). qRT-PCR analysis of osteogenesis-related genes further confirmed these findings (Supplementary Fig. 27). These results demonstrate that LNP@A significantly enhanced the osteogenic potential of aged BMSCs in vitro, which is consistent with our theoretical framework.

### 2.7 LNP@A demonstrates favorable biosafety after local administration

**Fig. 7:**
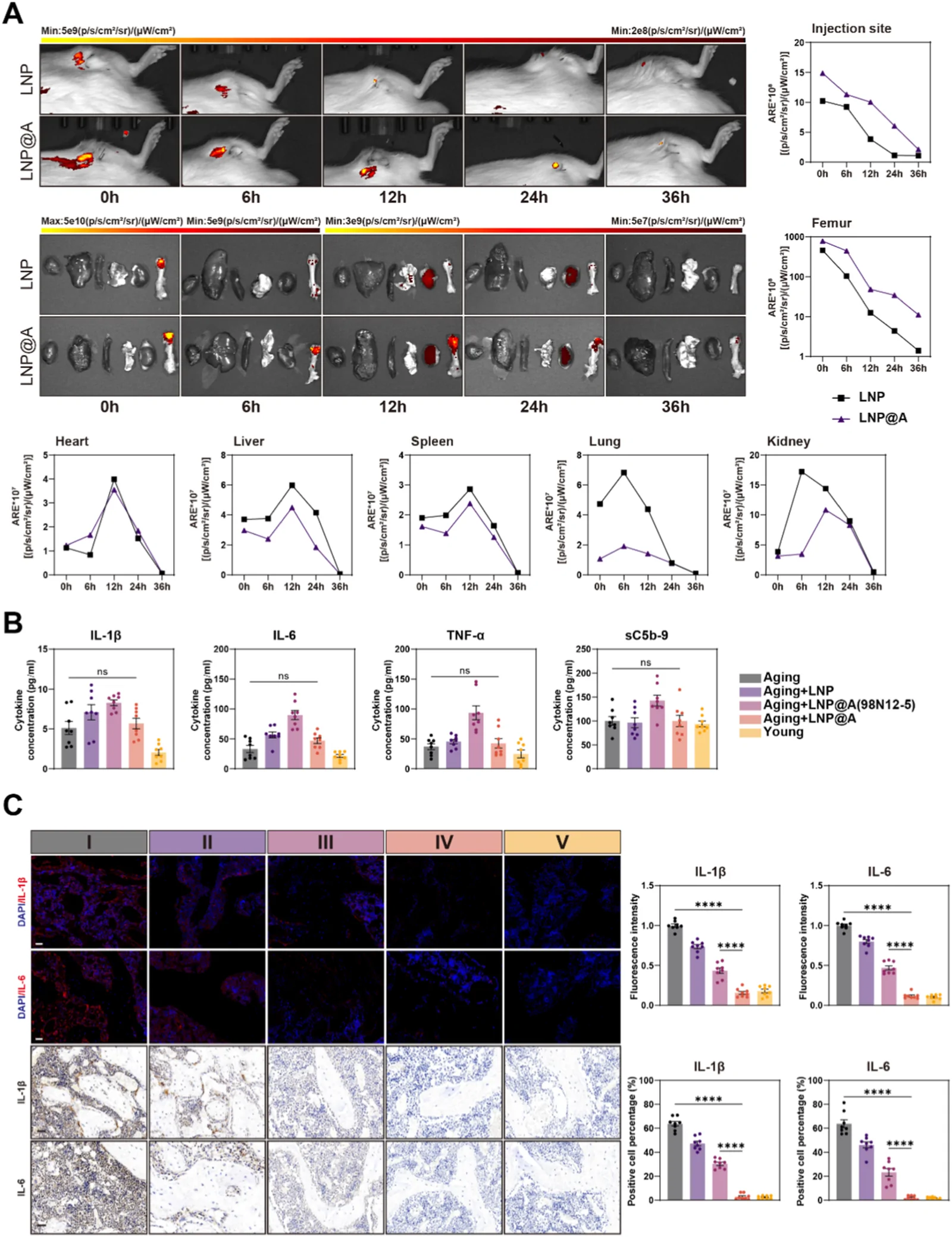
In vivo safety evaluation of LNP@A. **A.** In vivo fluorescence imaging analysis of LNP@A biodistribution following local injection and corresponding quantitative analysis (n = 8 biologically independent samples). **B.** ELISA quantification of serum inflammatory cytokine levels following local administration of LNP@A (n = 8 biologically independent samples). **C.** Representative immunofluorescence staining images and corresponding semi-quantitative analysis of inflammatory cytokine expression in rat bone tissue sections following local administration of LNP@A (n = 8 biologically independent samples). Data are presented as mean ± standard error of the mean (SEM). Statistical analysis was performed using two-tailed Student's t-test for comparisons between two groups and one-way analysis of variance (ANOVA) followed by Tukey's multiple comparisons test for comparisons among multiple groups. The number of biological replicates (n) is indicated in each figure legend. Statistical significance is indicated as *p < 0.05, **p < 0.01, and ***p < 0.001.

To characterize the in vivo behavior of LNP@A, Cy5-labeled LNP@A was locally injected into the bone defect site, and its biodistribution was monitored (Fig. 7A). Compared with non-targeted LNP controls, LNP@A demonstrated enhanced and sustained enrichment at the defect region, with significant retention maintained up to 36 h. Subsequently, the fluorescence signal was predominantly cleared through the renal pathway, and no detectable accumulation was observed in major organs, suggesting favorable in vivo clearance and biosafety (Fig. 7A and Supplementary Fig. 28). Furthermore, serum inflammatory cytokine analysis revealed that LNP@A did not trigger systemic inflammatory responses following local injection (Fig. 7B). Consistently, immunofluorescence and immunohistochemical analyses of the defect tissue showed that LNP@A significantly suppressed local IL-1β and IL-6 expression, indicating its potent local anti-inflammatory therapeutic effect (Fig. 7C).

### 2.8 LNP@A enhances the osteogenic potential of aged rats in vivo

**Fig. 8:**
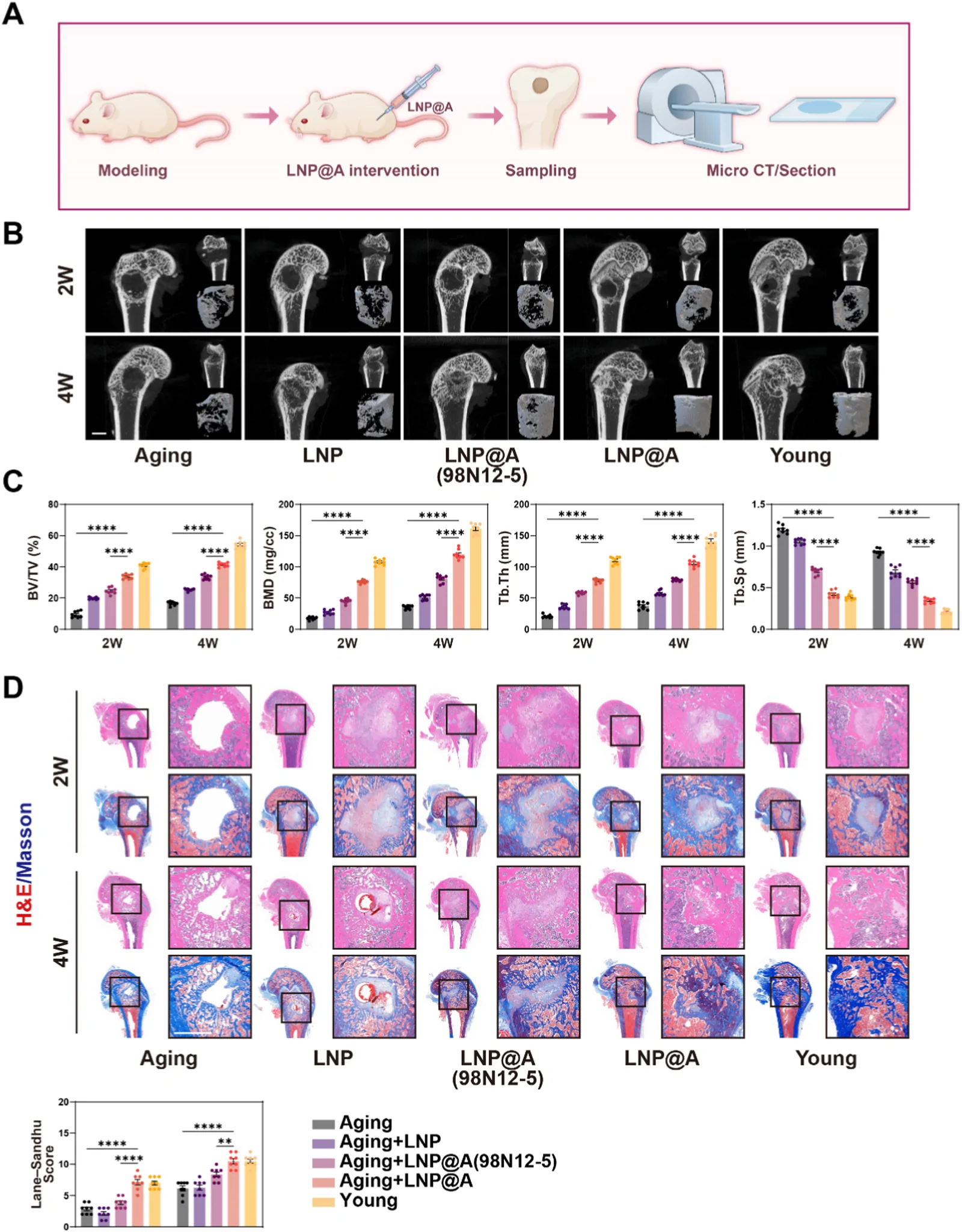
LNP@A enhances osteogenic potential in vivo. **A.** Schematic diagram of the animal experimental workflow. **B.** Micro-CT evaluation of the femoral condyle defect model at 2 and 4 weeks post-surgery following intervention with LNP without targeting peptide, LNP@A with 98N12-5, and LNP@A (scale bar = 1.5 mm). **C.** Quantitative analysis of BV/TV, BMD, Tb.Th, and Tb.Sp at 2 and 4 weeks post-surgery (n = 8 biologically independent samples). **D.** Representative H&E and Masson's trichrome stained images of femoral condyles at 2 and 4 weeks post-surgery (scale bar = 1.5 mm). **E.** Lanne–Sandhu scoring analysis of HE and Masson's trichrome staining (n = 8 biologically independent samples). Data are presented as mean ± standard error of the mean (SEM). Statistical analysis was performed using two-tailed Student's t-test for comparisons between two groups and one-way analysis of variance (ANOVA) followed by Tukey's multiple comparisons test for comparisons among multiple groups. The number of biological replicates (n) is indicated in each figure legend. Statistical significance is indicated as *p < 0.05, **p < 0.01, and ***p < 0.001. BMSCs: Bone marrow-derived mesenchymal stem cells; BV/TV: Bone volume/tissue volume; BMD: Bone mineral density; Tb.Th: Trabecular thickness; Th.Sp: Trabecular spacing.

We selected an aged rat femoral condyle defect model and locally injected LNP@A into the bone defect site for subsequent assessment (Fig. 8A). At 2 weeks, micro-computed tomography (micro-CT) in both the coronal and sagittal planes revealed increased new bone formation in the bone defect area of the LNP@A intervention group compared with the aged control group (Fig. 8B). Quantitative analysis (Fig. 8C) indicated that LNP@A improved bone volume/tissue volume (BV/TV), bone mineral density (BMD), trabecular thickness (Tb.Th), and trabecular spacing (Th.Sp), demonstrating its potent bone repair capacity. By week 4, no significant bone defect repair was observed in the aged group. In contrast, the bone defects in the young and LNP@A groups showed marked expansion and relative densification, with newly formed bone tissue filling nearly the entire defect area. Three-dimensional reconstructions of the defective regions exhibited similar trends (Fig. 8B). Additionally, quantitative analysis revealed that BV/TV, BMD, and Tb.Th in the LNP@A intervention group increased over time, whereas Th.Sp decreased (Fig. 8C). Additionally, we performed hematoxylin and eosin (H&E) staining and Marzon staining (Fig. 8D). At 2 weeks post-surgery, the bone defect area was filled with soft tissue, with more regular new bone tissue visible at the margins of the defect in the LNP@A group. At 4 weeks post-surgery, the bone defects in all the control groups remained unhealed, showing partial new bone formation around the periphery, while the center remained filled with soft tissue. In contrast, the LNP@A group exhibited significant healing, with dense new bone visible at the center. These findings indicate that the LNP@A intervention effectively enhanced the bone regenerative capacity in aged rats.

Given that bone defect repair may affect postoperative pain-associated behaviors, we performed behavioral assessments using the VisuGait gait analysis system and hindlimb weight distribution test. LNP@A-treated rats showed earlier recovery of hindlimb loading and increased weight-bearing on the affected limb compared with controls, suggesting reduced pain-related behaviors and confirming the therapeutic potential of LNP@A. In addition, mechanical compression testing of both the whole femoral condyle and newly formed bone further confirmed the improved quality and functional restoration of regenerated bone following LNP@A treatment (Supplementary Fig. 30).

**Fig. 9:**
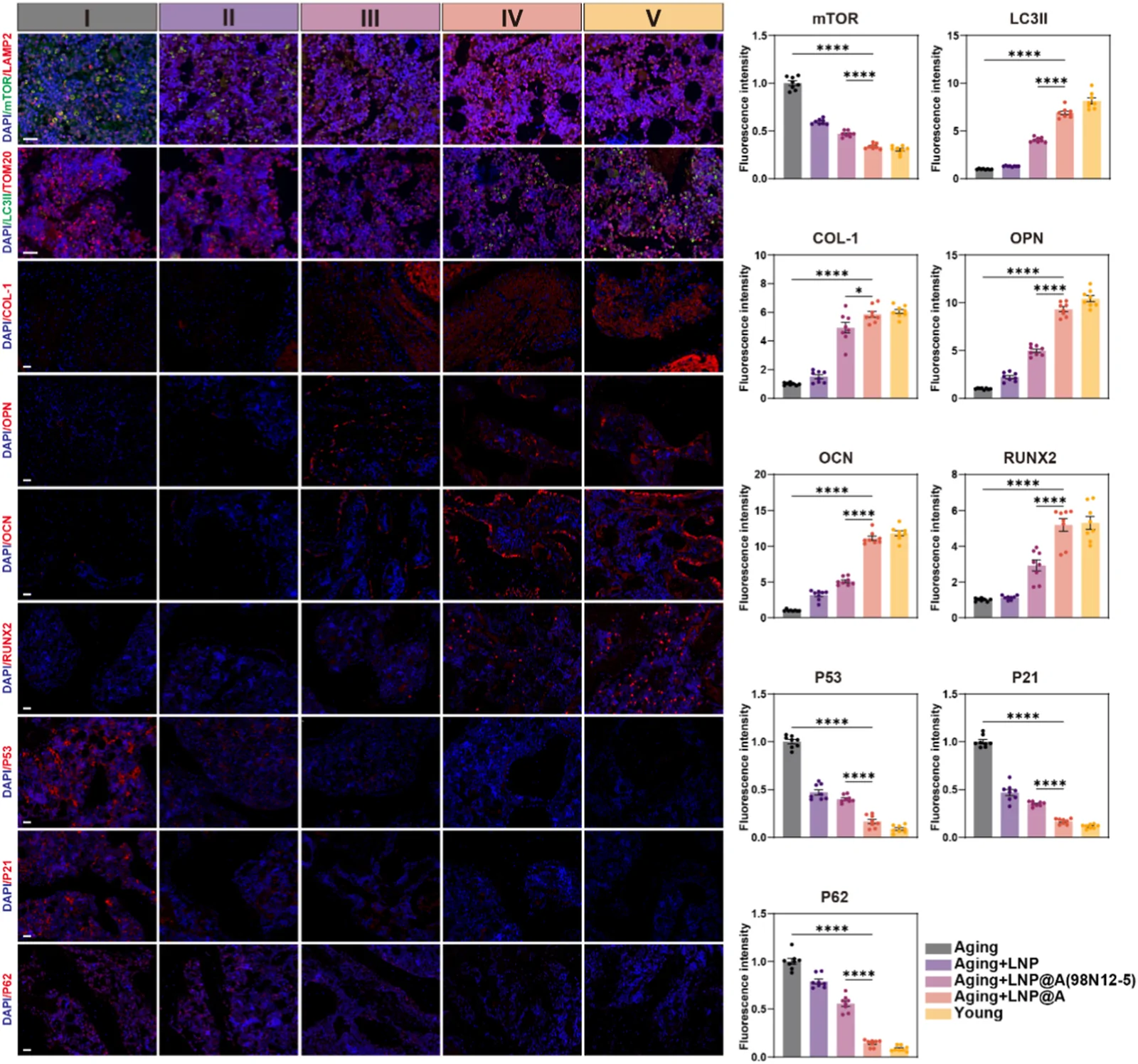
Representative immunofluorescence staining images (scale bar = 50 μm) and semi-quantitative analysis (n = 8 biologically independent samples) of autophagy-, osteogenesis-, and senescence-related markers in rat bone tissue sections following LNP@A treatment. Data are presented as mean ± standard error of the mean (SEM). Statistical analysis was performed using two-tailed Student's t-test for comparisons between two groups and one-way analysis of variance (ANOVA) followed by Tukey's multiple comparisons test for comparisons among multiple groups. The number of biological replicates (n) is indicated in each figure legend. Statistical significance is indicated as *p < 0.05, **p < 0.01, and ***p < 0.001.

**Fig. 10:**
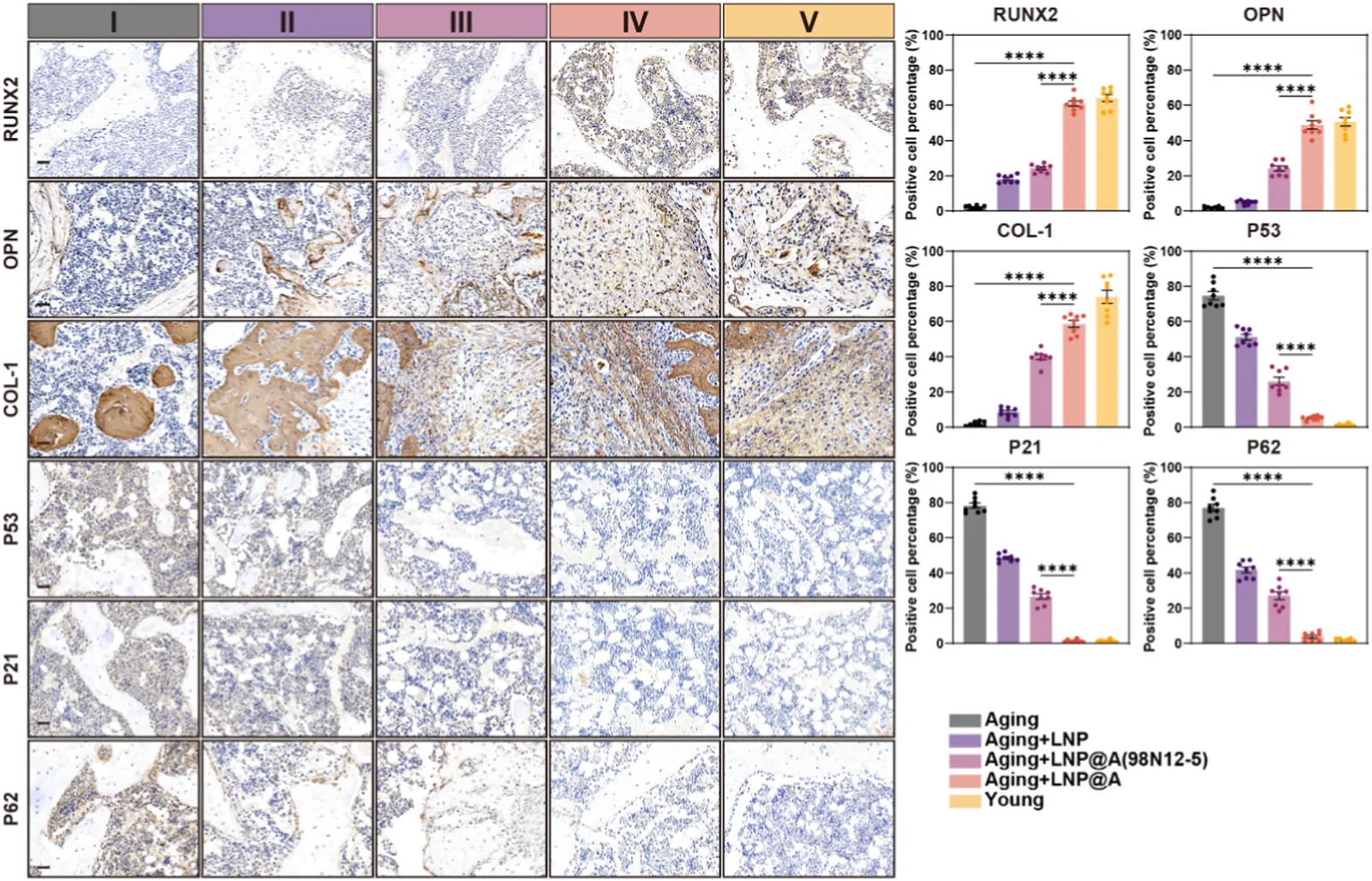
Representative immunohistochemical staining images (scale bar = 50 μm) and corresponding semi-quantitative analysis (n = 8 biologically independent samples) of autophagy-, osteogenesis-, and senescence-related markers in rat bone tissue sections following LNP@A treatment. Data are presented as mean ± standard error of the mean (SEM). Statistical analysis was performed using two-tailed Student's t-test for comparisons between two groups and one-way analysis of variance (ANOVA) followed by Tukey's multiple comparisons test for comparisons among multiple groups. The number of biological replicates (n) is indicated in each figure legend. Statistical significance is indicated as *p < 0.05, **p < 0.01, and ***p < 0.001.

Immunofluorescence and immunohistochemical analyses of bone sections revealed that LNP@A effectively reduced mTORC1 activity, restored autophagic activity, alleviated cellular senescence, and enhanced osteogenic potential in vivo (Fig. 9, Fig. 10). Furthermore, we isolated lysosomal fractions from bone tissues and quantified cholesterol levels (Supplementary Fig. 29). The results demonstrated that LNP@A markedly reduced lysosomal cholesterol accumulation, consistent with its ability to restore cholesterol homeostasis. These findings align with our in vitro conclusions that LNP@A enhances the osteogenic potential of aged BMSCs by activating autophagy via mTOR inhibition, regulating ABCA1 organelle distribution, and improving cellular senescence. Thus, we propose that LNP@A offers a novel therapeutic approach targeting organelle interactions to restore metabolic homeostasis, presenting translational prospects for interventions in age-related bone metabolic disorders.

Beyond bone regeneration, ABCA1 serves as a critical target in cardiovascular diseases, where ABCA1-mediated lipid efflux plays a protective role against atherosclerosis [73,74]. Similarly, ABCA1 has been implicated in the progression of numerous neurological disorders, including Alzheimer's and Huntington's disease [7,75]. Furthermore, ABCA1 has been implicated in the glomerular endothelial injury associated with diabetic nephropathy [76]. Given the pivotal role of ABCA1 in numerous physiological and pathological processes, we propose that the effects of RA and LNP@A may extend beyond the repair of age-related bone defects. Depending on the specific disease, modifying the targeting sequence of engineered enzymes, replacing the targeting peptide on the liposome surface, or altering the composition of the liposome could potentially exert positive effects on disease progression. Furthermore, our work offers a novel approach for diseases involving the subcellular distribution of palmitoylated proteins: by intervening in a specific step of the palmitoylation/depalmitoylation cycle through organelle-targeted strategies while largely preserving other steps, we can tilt the entire cycle toward our desired direction, thereby minimizing side effects.

However, this study has several limitations. First, although our data support a model in which depalmitoylation promotes the release of ABCA1 from the endolysosomal membrane and facilitates its recycling, we did not directly monitor the dynamic intracellular trafficking of ABCA1. Therefore, the proposed palmitoylation cycle is unlikely to represent the full complexity of ABCA1 trafficking, and alternative fates of endolysosomal ABCA1 cannot be excluded. For example, ABCA1 has been reported to undergo ubiquitination-dependent, ESCRT-mediated lysosomal degradation [74,77]. Because neither ubiquitination nor ESCRT activity was examined in the present study, our data cannot determine whether depalmitoylation competes with, precedes, or cooperates with this degradative pathway. Given the extensive crosstalk between palmitoylation and ubiquitination, whether palmitoylation influences ABCA1 ubiquitination and its subsequent ESCRT-dependent degradation warrants further investigation. In addition, although our findings suggest that depalmitoylation facilitates the recycling of ABCA1 to the Golgi apparatus, we did not identify the adaptor proteins responsible for this trafficking step. Consequently, the molecular machinery linking depalmitoylation to retrograde transport remains undefined. Previous studies have identified ARF6 as a regulator of plasma membrane ABCA1 recycling [78]. Whether palmitoylation modulates the interaction between ABCA1 and ARF6, thereby coordinating distinct trafficking pathways, also remains unknown. Addressing these questions will further refine the proposed model of ABCA1 trafficking and improve our understanding of how palmitoylation coordinates intracellular cholesterol homeostasis, ultimately facilitating the development of more precise therapeutic strategies.

Although our data support the lysosomal localization of RA based on subcellular colocalization, lysosomal fractionation, and its effects on ABCA1 redistribution, the dependence of this targeting on the RAB7 sequence was not directly examined. Future studies employing RAB7 loss-of-function mutants will be important to validate the targeting mechanism and exclude potential off-target effects. Our results support DHHC8 and APT2 as key regulators of ABCA1 palmitoylation and depalmitoylation and identify several major palmitoylation sites of ABCA1. However, the regulation of individual ABCA1 palmitoylation sites by DHHC8 or APT2 remains unclear and requires further investigation. Although our biodistribution analysis demonstrated the accumulation of LNP@A at the bone defect site, the contribution of different cell populations within the defect microenvironment remains to be further elucidated. LNP@A was designed to target uPAR-expressing senescent cells rather than a specific cell type. In this study, senescent BMSCs were identified as an important responder population because of their central role in osteogenic regeneration; however, other uPAR-expressing senescent cells may also contribute to the therapeutic effects of LNP@A. Cell-type-specific co-localization analysis combining cell-type markers with ABCA1, cholesterol signals, autophagy, and senescence markers remains technically challenging in decalcified bone tissues due to limitations in Filipin-based cholesterol visualization, multi-marker staining, and the spatial resolution of tissue immunofluorescence. Future studies using advanced spatial imaging approaches will further define the cellular mechanisms underlying LNP@A-mediated bone regeneration. Another limitation of this study is that additional controls, such as catalytic-dead RA and untargeted APT2 delivery, are needed to further dissect the contribution of APT2 enzymatic activity and lysosomal targeting to the observed effects. Future studies will further clarify the specificity and mechanism of this spatial regulation strategy. Finally, although RA was designed to achieve organelle-selective regulation by restricting APT2 activity to the lysosomal compartment, we cannot exclude potential effects on other lysosomal or plasma membrane palmitoylated proteins. The current study did not comprehensively evaluate global changes in protein palmitoylation or define the complete substrate spectrum of RA. Future studies integrating global palmitome profiling and systematic characterization of additional APT2 substrates will be required to further elucidate the specificity and broader biological effects of this organelle-targeted depalmitoylation strategy.

Overall, our study revealed the role of ABCA1-mediated lysosomal cholesterol accumulation in autophagy suppression in aged BMSCs and investigated how the palmitoylation cycle of ABCA1 affects its subcellular distribution. Based on this mechanism, we engineered the organelle-targeted depalmitoylase RA and employed a senescence-targeting lipid nanoparticle as the delivery vehicle for RA mRNA. LNP@A drives the palmitoylation cycle of ABCA1 toward the plasma membrane in aged BMSCs. Its efficacy in restoring autophagy to reverse cellular aging and ultimately enhance osteogenic potential was validated both in vitro and in vivo. Our study provides novel therapeutic insights for interventions in age-related bone defects and other diseases involving the protein palmitoylation/depalmitoylation cycles.

## 3 Methods

### 3.1 Cell culture and intervention

BMSCs were isolated from the bone marrow of 4-week-old SD rats. Under aseptic conditions, bone marrow was minced to dissociate BMSCs into small cell clusters. Cells were seeded in DMEM-α medium containing 10% fetal bovine serum (FBS) (F814-100, BaiDi, China) and 1% penicillin-streptomycin (Gibco, USA), and cultured in a 5% CO_2_ incubator at 37°C. Cells were passaged upon reaching 70% confluence. (12561056, Gibco, USA) supplemented with 10% fetal bovine serum (FBS) and 1% penicillin-streptomycin. Cells were cultured at 37°C in a 5% CO_2_ incubator. Passages were performed when cells reached 70% confluence. BMSCs were passaged to p12 to induce cellular senescence. Drugs were administered at the following concentrations: rapamycin (HY-10219, MCE, USA), 10 nM; chloroquine (HY-17589A, MCE, USA), 100 μM; 2-bromhexadecanoic acid (HY-111770, MCE, USA), 50 μM; palmostatin B (178501, Sigma, USA), 50 μM; ML348 (HY-100736, MCE, USA), 20 μM; ML349 (HY-100737, MCE, USA), 20 μM; Brefeldin A (HY-16592, MCE, USA), 20 μM.

### 3.2 Plasmid construction and transfection

Full-length cDNAs of rat ABCA1, DHHC8, and APT2 were amplified from cDNA libraries. Using Phanta Max Super-Fidelity DNA Polymerase (Vazyme, China), appropriate tags were ligated and cloned into the pLenti vector. For RAB7-APT2 targeting late endosomes and lysosomes, the RAB7 sequence was flexibly connected to the C-terminus of APT2. The sequence was synthesized as a gene fragment and cloned into the pLenti vector. All sequences used for transfection are detailed in Table S2. All constructs were validated by DNA sequencing to confirm plasmid sequences. Plasmid transfection was performed using Lipo8000™ Transfection Reagent (C0533, Beyotime, China) or Lipofectamine 3000 (L3000015, ThermoFisher, USA) according to manufacturer protocols.

### 3.3 Site-directed mutagenesis and rescue experiments

ABCA1 mutants carrying the C3S, C23S, C1110S, C1111S, or A937V mutation were generated by site-directed mutagenesis using the Mut Express II Fast Mutagenesis Kit V2 (Vazyme, Nanjing, China) according to the manufacturer's instructions. All mutant constructs were verified by Sanger sequencing before use. For rescue experiments, endogenous ABCA1 was first silenced using shRNA targeting ABCA1, followed by transfection of shRNA-resistant wild-type or mutant ABCA1 expression plasmids into the knockdown cells. Cells expressing wild-type or mutant ABCA1 were subsequently used for functional analyses.

### 3.4 Isolation and enrichment of organelles

For lysosomes, use the Lysosome Enrichment Kit (89839, ThermoFisher, USA) to isolate lysosomes according to the instructions for subsequent experiments. For plasma membranes, the Cell Surface Biotinylation and Isolation Kit (A44390, ThermoFisher, USA) was used according to the manufacturer's instructions to isolate cell surface proteins. For the Golgi apparatus, the Golgi Extraction Kit (EX1360, Solarbio, China) was employed following the protocol to enrich Golgi complexes.

### 3.5 Cholesterol depletion and stimulation

Cells were cultured for 2 days in medium containing 0.5% (w/v) MβCD (HY-101461, MCE, USA) to achieve cholesterol depletion. Cells were stimulated with cholesterol by treating them with 50 μM water-soluble cholesterol (HY-N0322A, MCE, USA).

### 3.6 Filipin staining

Seed cells into a 24-well plate, fix with paraformaldehyde for 10 min, then block with commercial rapid blocking solution (P0260, Beyotime Biotechnology, China) for 20 min. Incubate with LAMP2 antibody (GL2A7, ThermoFisher, USA) overnight at 4°C. Subsequently, add appropriate secondary antibody, DAPI (40728ES03, Yeasen, China), and filipin complex (HY-N6716, MCE, USA) at a concentration of 0.05 mg/ml. Incubate for 2 h. After each step, wash three times with PBS for 10 min each. Observe using a confocal microscope (Leica, Germany) and perform colocalization analysis with ImageJ.

### 3.7 Cholesterol quantification

Directly lyse cells and sonicate homogenates as required, or use the Lysosome Enrichment Kit (89839, ThermoFisher, USA) to extract lysosomes according to the instructions before proceeding with lysis and homogenization. Perform staining using the Amplex Red Cholesterol Assay Kit (A12216, ThermoFisher, USA) following the protocol. Quantify cholesterol concentration in samples using an Epoch microplate reader (BioTek, USA). Normalize cholesterol concentration to total cellular protein (P0012, Beyotime, China). Calculate relative cholesterol levels between groups by comparing normalized cholesterol levels.

### 3.8 Cholesterol efflux assay

Perform the Cholesterol Efflux Assay Kit (ab196985, Abcam, UK) according to the manufacturer's instructions. Briefly, incubate cells with fluorescent cholesterol, wash them, then measure the fluorescence intensity of both the supernatant and cell lysate using an Epoch microplate reader (BioTek, USA) to calculate the cholesterol efflux rate.

### 3.9 Transcriptomics

BMSCs collected from p3 and p12 were subjected to transcriptomic analysis. Total RNA was extracted using an RNA purification kit (ThermoFisher, USA). Differential analysis was performed using thresholds of FDR < 0.05 and log2FC > 1. Subsequently, GO and KEGG enrichment analyses were conducted to identify biological functions or pathways significantly altered during the aging process.

### 3.10 Senescence-associated β-galactosidase staining

Seed cells into 6-well or 12-well plates and culture to appropriate density. Use the Senescence β-Galactosidase Staining Kit (C0602, Beyotime, China) to perform fixation and prepare staining solutions according to the instructions. Cover with plastic wrap and incubate at 37°C overnight. Observe and image using an optical microscope.

### 3.11 Alkaline phosphatase (ALP) staining

After 7 days of cell culture, cells were first fixed with 4% paraformaldehyde for 10 min, washed three times with PBS for 10 min each, then stained using the Alkaline Phosphatase Stain Kit (40749ES60, Yeasen, China) according to the manufacturer's instructions. Stained cells were observed and imaged under a light microscope. Cells were lysed using RIPA buffer and stained with the Fluorometric Alkaline Phosphatase Assay Kit (P0322S, Beyotime, China) according to the manufacturer's instructions. Fluorescence intensity was measured using an Epoch microplate reader (BioTek, USA) to quantify ALP levels in the samples.

### 3.12 Alizarin red S (ARS) staining

After 14 days of cell culture, cells were first fixed with 4% paraformaldehyde for 10 min, followed by three washes with PBS (10 min each). Subsequently, cells were stained using the Alizarin Red S Staining Kit (C0148S, Beyotime, China) according to the manufacturer's instructions, and observed and imaged under a light microscope. Add 1 ml of 10% perchloric acid solution to each well and incubate at room temperature in the dark until calcium nodules are completely dissolved. Quantify ARS levels by measuring fluorescence intensity using an Epoch microplate reader (BioTek, USA).

### 3.13 Mitochondrial membrane potential assay

Prepare the staining solution using the Enhanced mitochondrial membrane potential assay kit with JC-1 (C2003S, Beyotime, China) according to the instructions. Incubate at 37°C for 20 min. Subsequently, wash twice with JC-1 buffer solution for 5 min each time. Then replace with culture medium and observe under a fluorescence microscope (Carl Zeiss, USA). Perform semi-quantitative analysis using ImageJ.

### 3.14 Autophagosome tracking

We selected Ad-mCherry-GFP-LC3B (C3011, Beyotime, China) to label autophagosomes and performed transfection using Lipofectamine 3000 (L3000015, ThermoFisher, USA). Observations and imaging were performed using confocal microscopy (Leica, Germany). Subsequently, autophagy bodies were counted by participants unaware of the experimental conditions, enabling semi-quantitative analysis.

### 3.15 Mitochondria-lysosome colocalization staining

Live cells were stained with Lysotracker Green (40738ES50, Yeasen, China) and Mitotracker Red (M7512, ThermoFisher, USA) by preparing working solutions according to the manufacturers' instructions. After incubating in the dark for 30 min, cells were washed three times with culture medium for 5 min each wash. Immediately thereafter, cells were observed and imaged using a confocal microscope (Leica, Germany).

### 3.16 Cellular immunofluorescence

Cells were seeded in 24-well plates and cultured to appropriate density. They were fixed with paraformaldehyde for 10 min, then blocked with commercial rapid blocking solution (P0260, Beyotime Biotechnology, China) for 20 min. The following primary antibodies were added: P21 (YM8364, Immunoway, USA), P53 (YT3528, Immunoway, USA), TOM20 (A27799, Abclonal, China), LC3II (A19665, Abclonal, China), mTOR (YM8208, Immunoway, USA), LAMP2 (GL2A7, ThermoFisher, USA), ABCA1 (A7228, Abclonal, China), HA (ab18181, Abcam, UK), uPAR (GTX638173-S, Genetex, USA), OPN (30200-1-AP, Proteintech, USA), RUNX2 (ab76956, Abcam, UK), and incubated overnight at 4°C. Add appropriate secondary antibodies as needed: Rhodamine Phalloidin (40734ES75, Yeasen, China), and DAPI (40728ES03, Yeasen, China). Incubate at 37°C in the dark for 2 h. Wash three times with PBS after each step, each wash lasting 10 min. Observe and image using a fluorescence microscope (Carl Zeiss, USA) or confocal microscope (Leica, Germany) as needed. Perform semi-quantitative analysis and colocalization analysis using ImageJ.

### 3.17 Immunoblotting

Following cellular intervention, total proteins were extracted using RIPA buffer (P0013B, Beyotime, China) supplemented with protease inhibitors (P1006, Beyotime, China) and phosphatase inhibitors (P1081, Beyotime, China), or using enriched organelles. Protein concentration was determined using the BCA assay kit (P0012, Beyotime Biotechnology, China). Equal amounts of protein were electrophoresed on SDS-PAGE and transferred to polyvinylidene fluoride (PVDF) membranes. Overnight incubation with the following primary antibodies was performed: LC3II (A19665, Abclonal, China), P62 (A19700, Abclonal, China), Parkin (A0968, Abclonal, China), Pink1 (A24745, Abclonal, China), β-actin (AC038, Abclonal, China), P-s6k (AP0502, Abclonal, China), S6k (A2190, Abclonal, China), P-mTOR (AP1413, Abclonal, China), mTOR (A11354, Abclonal, China), ABCA1 (A22125, Abclonal, China), LAMP2 (A26781PM, Abclonal, China), Na+/K + -ATPase (A7878, Abclonal, China), GM130 (A11408, Abclonal, China), HA (ab18181, Abcam, UK), P16 (A23882, Abclonal, China), P21 (A19094, Abclonal, China), P53 (A19585, Abclonal, China), IL-1β (A23484, Abclonal, China), IL-6 (A0268, Abclonal, China), RUNX2 (A2851, Abclonal, China), OPN (30200-1-AP, Proteintech, USA), and OCN (A6205, Abclonal, China). The membranes were then incubated with horseradish peroxidase (HRP)-conjugated secondary antibody (A0208, Beyotime, China). Band detection was performed using the ECL kit (E422, Vazyme, China), and visualized with a gel imaging system.

### 3.18 Co-immunoprecipitation

For immunoprecipitation, cells were re-treated and lysed using RIPA buffer. A portion of the lysate served as input, while the remaining lysate was incubated overnight at 4°C with the following primary antibodies: ABCA1 (A22125, Abclonal, China), APT2 (sc-390546, Santa Cruz, USA), and incubated overnight at 4°C with A/G magnetic beads (P2108, Beyotime, China) or IgG magnetic beads (Beyotime, China). After five washes, the protein was eluted using SDS-PAGE loading buffer (P0015A, Beyotime, China) for Western blotting.

### 3.19 ABE assay

The ABE assay was conducted largely in accordance with previous studies [76,79].。In brief, add 20 mM N-ethylmaleimide (HY-D0843, MCE, USA) to the cell lysate and incubate at 4°C for 1.5 h. Centrifuge, collect the supernatant, and quantify it. Precipitate proteins using a methanol-chloroform-water mixture. Resuspend proteins in buffer. Divide into two groups based on whether HAM (H828371, MACKLIN, China) is added. Incubate at room temperature with gentle rotation for 1 h, then precipitate proteins again. Resuspend proteins in buffer once more, add 4 mM Biotin-HPDP, and incubate at room temperature with gentle rotation for 1 h. Precipitate proteins again. Resuspend proteins in buffer and incubate with streptavidin magnetic beads (P2151, Beyotime, China) overnight at 4°C. Elute proteins using SDS-PAGE loading buffer (P0015A, Beyotime, China) for Western blotting.

### 3.20 qRT-PCR

Total RNA was extracted using the RNA Extraction Kit (R0018S, Beyotime, China). The concentration of RNA in each group was measured and normalized. Complementary DNA was synthesized using the HiScript III kit (R312, Vazyme, China) according to the manufacturer's instructions. Prepare qRT-PCR reaction mixtures. After denaturation, annealing, and extension, analyze target gene expression using glyceraldehyde-3-phosphate dehydrogenase (GAPDH) as an internal control. All primer sequences are listed in Table S1.

### 3.21 Preparation and characterization of LNP@A

mRNA was provided by Tsingke Biotechnology Co., Ltd. (Beijing, China), and AE105 was synthesized by Hefei Taikubio Co., LTD (Hefei, China). The mRNA sequence of RA is provided in Table S3. First, we covalently linked DMG-PEG2000-NHS (M59171, AbMole, USA) to AE-105 using EDC chemistry. The formation of the covalent bond was confirmed by infrared spectroscopy, yielding DMG-PEG2000-AE105. Following a previously established method [63,64], we mixed 4A3-SC8 (HY-148559, MCE, USA), DOPE (HY-112005, MCE, USA), cholesterol (ST1155, Beyotime, China), and our synthesized DMG-PEG2000-AE105 were dissolved in ethanol as the ethanol phase at a molar ratio of 50/10/38.5/1.5. RA mRNA was dissolved in citrate buffer (pH = 4) as the aqueous phase. with a flow rate ratio of 3:1 between the aqueous and ethanol phases and a mass ratio of mRNA to total lipids of 1:40. The aqueous and ethanol phases were mixed using microfluidic technology, followed by dialysis to obtain the targeted liposome LNP@A carrying RA mRNA. LNP without the targeting peptide was synthesized using 98N12-5 (HY-112772A, MCE, USA), cKK-E12 (HY-134781, MCE, USA), and C12-200 (HY-145405, MCE, USA). For Cy5-fluorescently labeled LNP and LNP@A, DOPE-Cy5 was substituted for DOPE in synthesis. The DOPE-Cy5 used was purchased from Xi'an Qiyue Biotechnology Co., Ltd. The successful loading of mRNA onto LNP@A was verified via agarose gel electrophoresis. One gram of agarose was dissolved in 100 ml TAE buffer and heated in a microwave until completely dissolved. Samples were mixed with markers and GalGreen (G5570, Solarbio, China), followed by electrophoresis and visualization using a gel imaging system. Dynamic light scattering (Malvern, UK) was employed to determine LNP@A's particle size, polydispersity index (PDI), and zeta potential. The LNP encapsulation efficiency was quantified using the Quant-it RiboGreen RNA Assay Kit (R11490, ThermoFisher, USA). Transmission electron microscopy (TEM, Carl Zeiss, USA) confirmed the synthesis of LNP@A.

### 3.22 Cell adhesion and proliferation

Seed cells into a 96-well plate and add LNP@A to the medium at concentrations of 0.75 μg/ml, 1.5 μg/ml, 3 μg/ml, and 6 μg/ml. Incubate at 37°C, 95% relative humidity, and 5% CO_2_. On days 1, 3, and 5, replace the medium with a solution containing 10% CCK-8 (C0038, Beyotime, China). Incubate for 1 h and measure absorbance at 450 nm using an Epoch microplate reader (BioTek, USA).

### 3.23 Live/dead cell staining

Based on the results of the CCK8 assay, a concentration of 3 μg/ml was selected for treatment at 1, 3, and 5 days. Cells were stained using the Calcein/PI Cell Viability/Cytotoxicity Assay Kit (C2015S, Beyotime, China) according to the manufacturer's instructions. Observations and imaging were performed using a fluorescence microscope (Carl Zeiss, USA).

### 3.24 Fluorescent liposome endocytosis

Cy5-labeled fluorescent LNP and LNP@A were incubated in culture medium for 24 h, then observed and imaged using a fluorescence microscope (Carl Zeiss, USA).

### 3.25 Animal preparation and modeling

Four-week-old and 12-month-old female Sprague–Dawley (SD) rats were obtained from the Experimental Animal Center of Soochow University. Animals were randomly assigned to the indicated experimental groups (n = 8 per group based on sample size calculation). A cylindrical femoral condyle defect (3 mm in diameter and 4 mm in depth) was surgically created under anesthesia. The femoral condyle defect used in this study (3.0 mm in diameter and 4.0 mm in depth) is below the critical-size threshold reported in recent studies and therefore retains a certain capacity for spontaneous bone regeneration. Accordingly, PBS-treated young and aged rats were included as control groups to evaluate the therapeutic efficacy of LNP@A in restoring age-impaired bone regeneration. Following surgery, rats received local injections of LNP@A into the femoral condyle defect site once weekly for a total of four administrations. Each injection was performed in a volume of 30 μL, containing 600 μg of total lipid and 15 μg of RA mRNA. The mRNA-to-total lipid mass ratio was maintained at 1:40. At 2 and 4 weeks post-surgery, rats were euthanized, and femoral specimens were harvested and fixed in 10% formalin solution. Micro-computed tomography (micro-CT; SkyScan 1276, Bruker, Germany) was performed to evaluate bone regeneration in the femoral condyle defect region. Three-dimensional reconstruction of the defect site was generated, and quantitative analysis was performed for bone regeneration-related parameters, including bone volume/tissue volume (BV/TV), bone mineral density (BMD), trabecular thickness (Tb.Th), and trabecular separation (Tb.Sp). Investigators responsible for micro-CT scanning and quantitative analysis were blinded to group allocation to minimize potential bias.

### 3.26 In vivo fluorescence imaging

To evaluate the in vivo biodistribution of LNP@A, nanoparticles were labeled with Cy5 prior to administration. Cy5-labeled LNP@A were locally injected into the femoral condyle defect. Fluorescence images were acquired at the indicated time points using an IVIS Spectrum in vivo imaging system (PerkinElmer, USA). At the end of the experiment, rats were euthanized, and the heart, liver, spleen, lung, kidney, and femurs were collected for ex vivo fluorescence imaging. Fluorescence intensity was quantified using Living Image software (PerkinElmer, USA).

### 3.27 Micro-CT

At 2 weeks and 4 weeks post-surgery, rats were euthanized, and femur specimens were harvested and fixed in 10% formalin solution. Micro-CT (SkyScan1276, Bruker, Germany) was used to image the femoral condyle region. Subsequently, 3D reconstruction of the bone defect site was performed, and the following parameters were analyzed: bone volume/tissue volume (BV/TV), bone mineral density (BMD), trabecular thickness (Tb.Th), and trabecular separation (Th.Sp).

### 3.28 Histological analysis of major organs

At the end of the experiment, the heart, liver, spleen, lung, and kidney were harvested, fixed in 4% paraformaldehyde for 24 h, dehydrated through a graded ethanol series, embedded in paraffin, and sectioned at a thickness of 5 μm. The sections were stained with hematoxylin and eosin (H&E) according to standard protocols and examined under a light microscope (Olympus, Japan) to evaluate potential histopathological changes.

### 3.29 HE and Masson's trichrome staining of tissue sections

Femora were decalcified in EDTA for 2 weeks, dehydrated in graded ethanol, cleared in xylene, and paraffin-embedded. Sections (5 μm) were deparaffinized in xylene, rehydrated through absolute and graded ethanol, and rinsed in distilled water.For H&E staining, sections were stained with hematoxylin for 5 min, differentiated in 1% HCl in 70% ethanol for 1 min, blued in ammonia water (pH 8.7) for 5 min, and counterstained with 0.5% eosin for 5 min. Sections were dehydrated in graded ethanol, incubated in 100% ethanol for 10 min, cleared in xylene for 30 min, mounted, and imaged by light microscopy.For Masson's trichrome staining, sections were stained with Masson's solution for 5 min, rinsed with 0.2% acetic acid, incubated in 5% phosphotungstic acid for 10 min, and washed twice with 0.2% acetic acid. Sections were then stained with aniline blue for 5 min, washed, dehydrated in absolute ethanol, cleared in xylene, mounted, and imaged by light microscopy.

### 3.30 Immunohistochemical staining

Femoral condyle specimens were fixed in 10% neutral buffered formalin, decalcified with 10% EDTA, embedded in paraffin, and sectioned at 5 μm thickness. Sections were deparaffinized, rehydrated, subjected to antigen retrieval, and incubated with primary antibodies overnight at 4°C. After incubation with secondary antibodies, signals were developed using a DAB substrate kit and counterstained with hematoxylin. Histological regeneration was quantitatively assessed using the Lanne–Sandhu scoring system, which evaluates bone defect repair based on parameters including the extent of new bone formation, quality of regenerated tissue, and defect bridging. Each section was independently scored by two observers who were blinded to the treatment groups, and the average score was used for statistical analysis.

### 3.31 Immunofluorescence staining of tissue sections

After fixation and dehydration, add 100 μL non-immunized goat serum and incubate at 37°C for 20 min. Use the following primary antibodies: mTOR, LAMP2, LC3II, TOM20, OPN, P21. Incubate overnight at 4°C. Then, incubate at 37°C for 30 min, add 100 μL of labeled fluorescent secondary antibody, incubate at 37°C for 1 h, wash three times with PBS, and finally mount sections with Fluoroshield containing DAPI.

### 3.32 Gait analysis

Gait analysis was performed using the VisuGait automated gait analysis system (Shanghai Xinruan Information Technology Co., Ltd., Shanghai, China). Before testing, rats were allowed to acclimate to the apparatus. Animals were encouraged to traverse the illuminated glass walkway at a consistent speed, and three compliant runs were recorded for each rat. Gait parameters were analyzed using VisuGait software according to the manufacturer's instructions.

### 3.33 Hindlimb weight-bearing assessment

Hindlimb weight-bearing was assessed using an incapacitance meter (Bioseb, France). Rats were placed in the testing chamber with each hindlimb positioned on an independent force plate. The weight borne by each hindlimb was recorded over three consecutive measurements, and the average value was used for statistical analysis.

### 3.34 Mechanical testing of the femoral condyle

The structural mechanical properties of the repaired femoral condyle were evaluated using a universal testing machine. After removal of the surrounding soft tissues, the femoral specimens were securely positioned on the testing platform, and a flat-ended cylindrical indenter with a diameter of 3 mm was aligned perpendicular to the defect area. Following preload, compression was applied at a constant displacement rate until a displacement of 2 mm was reached, which was defined as structural failure.

### 3.35 Mechanical testing of newly formed bone

The mechanical properties of the newly formed bone within the defect region were evaluated using a universal testing machine. A flat-ended cylindrical indenter with a diameter of 1.5 mm was carefully aligned with the center of the defect region. After application of a preload to ensure stable contact between the indenter and the specimen, compression was performed to a displacement of 0.5 mm.

### 3.36 Serum cytokine and complement analysis

Blood samples were collected from rats after LNP@A treatment and centrifuged to obtain serum. The levels of inflammatory cytokines, including IL-1β, IL-6, and TNF-α, as well as complement activation markers, were measured using commercially available enzyme-linked immunosorbent assay (ELISA) kits (Thermo Fisher Scientific, USA) according to the manufacturers’ instructions. Briefly, serum samples and standards were added to pre-coated ELISA plates and incubated according to the recommended protocols. After sequential incubation with detection antibodies and substrate solution, the absorbance was measured using a microplate reader. Cytokine and complement levels were calculated based on the corresponding standard curves.

### 3.37 Data analysis

Statistical analysis was performed using GraphPad Prism 10, Origin 9.1, and Microsoft Excel, with co-localization analysis conducted using ImageJ. Data are presented as mean ± s.e.m. Normality of data distribution was assessed using the Shapiro–Wilk test, and homogeneity of variance was evaluated using the Brown–Forsythe test before statistical analysis. Comparisons between two groups were performed using two-tailed unpaired Student's t-tests. Comparisons among multiple groups were performed using one-way analysis of variance (ANOVA) followed by Tukey's multiple comparisons test. A p value < 0.05 was considered statistically significant. Biological replicates were used for statistical analysis, and technical replicates were averaged before analysis.

All statistical analyses were performed using biological replicates as independent experimental units. For cell-based experiments, each biological replicate represented an independent cell culture experiment performed on a different occasion, and technical replicates within each biological replicate were averaged before statistical analysis. For animal experiments, each individual animal was considered one biological replicate. For microscopy-based analyses, five randomly selected, non-overlapping fields were quantified for each sample. For in vitro experiments, each field contained at least 10 cells. Data obtained from the five fields within each biological replicate were averaged, and the resulting mean value was used as a single independent data point for statistical analysis. The number of biological replicates (n) for each experiment is indicated in the corresponding figure legends.

## CRediT authorship contribution statement

**Mengze Cheng:** Investigation, Methodology, Visualization, Writing – original draft. **Kun Xi:** Visualization. **Zhengxia Ni:** Conceptualization, Visualization, Writing – original draft. **Lingjun Wang:** Methodology, Visualization. **Jincheng Tang:** Conceptualization. **Wei Wang:** Supervision. **Ziang Li:** Methodology. **Liang Zhou:** Conceptualization. **Xinzhao Jiang:** Conceptualization. **Jie Wu:** Formal analysis. **Qiangqiang Guo:** Investigation. **Yiwei Zhu:** Methodology. **Zhengjie Tao:** Investigation. **Tianyi Li:** Investigation. **Changdong Cui:** Investigation. **Wenguo Cui:** Supervision, Writing – review & editing. **Liang Chen:** Supervision, Writing – review & editing. **Yong Gu:** Supervision, Writing – review & editing.

## Ethics approval and consent to participate

All surgical procedures and perioperative management during the experiment were approved by the Ethics Committee of Soochow University (SUDA20251118A04).

## Declaration of competing interests

The authors declare that they have no competing interests.
