# Supplementary material for: A dual-targeting mRNA lipid nanoparticle restores ABCA1-dependent cholesterol homeostasis to promote repair of age-related bone defects
Source: Bioact Mater. 2026 Aug 20;67:620–42. doi: 10.1016/j.bioactmat.2026.08.017 (PMC13524491; doi:10.1016/j.bioactmat.2026.08.017)
Supplement: Supplementary file 1 [file mmc1.docx]

**A Dual-targeting mRNA Lipid Nanoparticle Restores ABCA1-dependent Cholesterol Homeostasis to Promote Repair of Age-related Bone Defects**

**Supplementary Figures**

**Supplementary Fig 1. A.** Representative immunofluorescence staining images of p53 in aged BMSCs following cholesterol depletion or stimulation (scale bar = 10μm) and semi-quantitative analysis (n=3 biologically independent samples). **B.** Quantitative PCR analysis of SASP inflammatory factors in aged BMSCs following cholesterol depletion or stimulation (n=3 biologically independent samples). Data are presented as mean ± standard error of the mean (SEM). Statistical analysis was performed using two-tailed Student’s t-test for comparisons between two groups and one-way analysis of variance (ANOVA) followed by Tukey’s multiple comparisons test for comparisons among multiple groups. The number of biological replicates (n) is indicated in each figure legend. Statistical significance is indicated as *p < 0.05, **p < 0.01, and ***p < 0.001.

**Supplementary Fig 2. A.** Western blot analysis of autophagy levels in young or aged BMSCs. **B.** Representative confocal images of LC3II and TOM20 staining in young or aged BMSCs, along with representative confocal images of Lysotracker and Mitotracker, and representative fluorescence images of JC-1 mitochondrial membrane potential (scale bar = 10μm). Colocalization analysis of LC3II and TOM20 staining with Lysotracker and Mitotracker staining using ImageJ (n=3 independent biological samples); ImageJ was used for semi-quantitative analysis of JC-1 red and green fluorescence intensities and calculation of the JC-1 ratio (n=3 biologically independent samples). Data are presented as mean ± standard error of the mean (SEM). Statistical analysis was performed using two-tailed Student’s t-test for comparisons between two groups and one-way analysis of variance (ANOVA) followed by Tukey’s multiple comparisons test for comparisons among multiple groups. The number of biological replicates (n) is indicated in each figure legend. Statistical significance is indicated as *p < 0.05, **p < 0.01, and ***p < 0.001.

**Supplementary Fig 3. A.** Representative fluorescence images of JC-1 mitochondrial membrane potential in BMSCs after cholesterol depletion or stimulation (scale bar = 10μm) and semi-quantitative analysis (n=3 biologically independent samples). **B.** Quantitative PCR analysis of SASP inflammatory factors in aged BMSCs after cholesterol depletion or stimulation (n=3 biologically independent samples). Data are presented as mean ± standard error of the mean (SEM). Statistical analysis was performed using two-tailed Student’s t-test for comparisons between two groups and one-way analysis of variance (ANOVA) followed by Tukey’s multiple comparisons test for comparisons among multiple groups. The number of biological replicates (n) is indicated in each figure legend. Statistical significance is indicated as *p < 0.05, **p < 0.01, and ***p < 0.001.

**Supplementary Fig 4. A.** Representative immunofluorescence staining images of p53 in BMSCs after cholesterol depletion or stimulation (scale bar = 10μm) and semi-quantitative analysis (n=3 biologically independent samples). **B.** Representative images of ARS and ALP staining in aged BMSCs after cholesterol depletion or stimulation (scale bar = 20 μm). Quantitative analysis of ARS and ALP staining (n = 11 independent biological samples). Data are presented as mean ± standard error of the mean (SEM). Statistical analysis was performed using two-tailed Student’s t-test for comparisons between two groups and one-way analysis of variance (ANOVA) followed by Tukey’s multiple comparisons test for comparisons among multiple groups. The number of biological replicates (n) is indicated in each figure legend. Statistical significance is indicated as *p < 0.05, **p < 0.01, and ***p < 0.001.

**Supplementary Fig 5. A.** Heatmap of differentially expressed genes (DEGs) between young and aged BMSCs. **B.** Volcano plot of DEGs. **C.** KEGG enrichment analysis of transcriptomics.

**Supplementary Fig 6. A.** Quantitative PCR analysis of ABCA1, ABCG1, and ABCA5 in aged BMSCs (n=3 biologically independent samples). **B.** Representative confocal images of mCherry-GFP-LC3-labeled autophagosomes following disruption of ABCG1 or ABCA5 expression (scale bar = 10μm) and semi-quantitative analysis (n=11 biologically independent samples). **C.** Representative confocal images of LC3II and TOM20 staining, representative confocal images of Lysotracker and Mitotracker, and representative fluorescence images of JC-1 mitochondrial membrane potential following disruption of ABCA1 expression (scale bar = 10μm), alongside their co-localization analysis and semi-quantitative analysis (n=3 independent biological samples). Data are presented as mean ± standard error of the mean (SEM). Statistical analysis was performed using two-tailed Student’s t-test for comparisons between two groups and one-way analysis of variance (ANOVA) followed by Tukey’s multiple comparisons test for comparisons among multiple groups. The number of biological replicates (n) is indicated in each figure legend. Statistical significance is indicated as *p < 0.05, **p < 0.01, and ***p < 0.001.

**Supplementary Fig 7.** Semi-quantitative analysis of mCherry-GFP-LC3-labeled autophagosomes (n=11 independent biological samples), colocalization analysis of LC3II/TOM20 staining (n=3 independent biological samples), colocalization analysis of Lysotracker/Mitotracker staining (n=3 independent biological samples), and semi-quantitative analysis of JC-1 mitochondrial membrane potential (n=3 independent biological samples) following ABCA1 expression disruption and complementation. Data are presented as mean ± standard error of the mean (SEM). Statistical analysis was performed using two-tailed Student’s t-test for comparisons between two groups and one-way analysis of variance (ANOVA) followed by Tukey’s multiple comparisons test for comparisons among multiple groups. The number of biological replicates (n) is indicated in each figure legend. Statistical significance is indicated as *p < 0.05, **p < 0.01, and ***p < 0.001.

**Supplementary Fig 8.** ABE assay to detect the effect of DHHC8 overexpression on ABCA1 palmitoylation levels.

**Supplementary Fig 9.** Western blot analysis of the effect of overexpressed palmitoyl transferase DHHC8 on lysosomal ABCA1 and plasma membrane ABCA1.

**Supplementary Fig 10.** Western blot analysis of ABCA1 subcellular distribution among different organelle fractions following APT2 knockdown and subsequent rescue by APT2 overexpression.

**Supplementary Fig 11. A.** Prediction of palmitoylation sites in rat and human ABCA1 using CSS-PALM 4.0, revealing high-scoring sites consistent with previously reported palmitoylation sites. **B.** ABE assay analysis of the effects of palmitoylation site mutations on ABCA1 palmitoylation levels. **C.** Western blot analysis of the effects of ABCA1 palmitoylation site mutations on mTORC1 activity.

**Supplementary Fig 12.** Representative confocal images (scale bar = 10μm) and semi-quantitative analysis of mCherry-GFP-LC3-labeled autophagosomes following cholesterol depletion or APT2 overexpression during ABCA1 rescue (n=11 biologically independent samples). Data are presented as mean ± standard error of the mean (SEM). Statistical analysis was performed using two-tailed Student’s t-test for comparisons between two groups and one-way analysis of variance (ANOVA) followed by Tukey’s multiple comparisons test for comparisons among multiple groups. The number of biological replicates (n) is indicated in each figure legend. Statistical significance is indicated as *p < 0.05, **p < 0.01, and ***p < 0.001.

**Supplementary Fig 13.** Detection of the effect of RA expression on the palmitoylation level of ABCA1 using the ABE method

**Supplementary Fig 14.** **A.** Co-localization analysis of ABCA1 with lysosomal staining and Filipin with lysosomal staining after expression of APT2 or RA (n=3 biologically independent samples). Semi-quantitative analysis of mCherry-GFP-LC3-labeled autophagosomes (n=11 independent biological replicates), colocalization analysis of LC3II and TOM20 staining with Lysotracker and Mitotracker staining (n=3 independent biological replicates), and semi-quantitative analysis of JC-1 mitochondrial membrane potential after APT2 or RA expression (n=3 biologically independent samples). **B.** Western blot analysis of ABCA1 subcellular distribution after RAB7 overexpression alone in BMSCs. **C.** Western blot analysis of autophagy-related protein expression following RAB7, APT2, or RA overexpression in BMSCs. Data are presented as mean ± standard error of the mean (SEM). Statistical analysis was performed using two-tailed Student’s t-test for comparisons between two groups and one-way analysis of variance (ANOVA) followed by Tukey’s multiple comparisons test for comparisons among multiple groups. The number of biological replicates (n) is indicated in each figure legend. Statistical significance is indicated as *p < 0.05, **p < 0.01, and ***p < 0.001.

**Supplementary Fig 15. A.** Quantitative analysis of SA-β-gal staining after expression of APT2 or RA (n=3 biologically independent samples). **B.** Representative immunofluorescence staining images of p53/p21 (scale bar = 10μm) and semi-quantitative analysis after expression of APT2 or RA (n=3 biologically independent samples). **C.** Quantitative PCR analysis of SASP inflammatory factors after APT2 or RA expression (n=3 biologically independent samples). Data are presented as mean ± standard error of the mean (SEM). Statistical analysis was performed using two-tailed Student’s t-test for comparisons between two groups and one-way analysis of variance (ANOVA) followed by Tukey’s multiple comparisons test for comparisons among multiple groups. The number of biological replicates (n) is indicated in each figure legend. Statistical significance is indicated as *p < 0.05, **p < 0.01, and ***p < 0.001.

**Supplementary Fig 16. A.** Semi-quantitative analysis of SA-β-gal-positive cells in BMSCs following RA treatment in the presence of ABCA1 knockdown (n=3 biologically independent samples). **B.** Representative immunofluorescence staining images (scale bar = 10μm) and corresponding semi-quantitative analysis (n=3 biologically independent samples) of p21 expression in BMSCs following RA treatment with or without ABCA1 knockdown. **C.** Representative immunofluorescence staining images (scale bar = 10μm) and corresponding semi-quantitative analysis (n=3 biologically independent samples) of p53 expression in BMSCs following RA treatment with or without ABCA1 knockdown. Data are presented as mean ± standard error of the mean (SEM). Statistical analysis was performed using two-tailed Student’s t-test for comparisons between two groups and one-way analysis of variance (ANOVA) followed by Tukey’s multiple comparisons test for comparisons among multiple groups. The number of biological replicates (n) is indicated in each figure legend. Statistical significance is indicated as *p < 0.05, **p < 0.01, and ***p < 0.001.

**Supplementary Fig 17. A.** Representative immunofluorescence staining images of p53 (scale bar = 10μm) and semi-quantitative analysis (n=3 biologically independent samples) after expressing RA while blocking autophagy with CQ. **B.** Quantitative PCR analysis of SASP inflammatory factors after expressing RA while blocking autophagy with CQ (n=3 biologically independent samples). Data are presented as mean ± standard error of the mean (SEM). Statistical analysis was performed using two-tailed Student’s t-test for comparisons between two groups and one-way analysis of variance (ANOVA) followed by Tukey’s multiple comparisons test for comparisons among multiple groups. The number of biological replicates (n) is indicated in each figure legend. Statistical significance is indicated as *p < 0.05, **p < 0.01, and ***p < 0.001.

**Supplementary Fig 18.** Time-course analysis of autophagic flux and autophagy-related protein expression by confocal imaging (scale bar = 10μm) and Western blotting, respectively.

**Supplementary Fig 19. A.** Assessment of cellular cholesterol levels and lysosomal cholesterol accumulation in young and aged primary BMSCs following RA treatment (n=3 biologically independent samples). **B.** Western blot analysis of RA-induced changes in the subcellular distribution of ABCA1 among different organelle fractions in primary BMSCs isolated from young and aged rats. **C.** Western blot analysis of mTORC1 activity and autophagy-related protein expression in RA-treated primary BMSCs isolated from young and aged rats. **D.** Representative SA-β-gal staining and immunofluorescence images of p21 and p53 expression (scale bar = 10μm), together with corresponding semi-quantitative analysis (n=3 biologically independent samples), in RA-treated primary BMSCs derived from young and aged rats. Data are presented as mean ± standard error of the mean (SEM). Statistical analysis was performed using two-tailed Student’s t-test for comparisons between two groups and one-way analysis of variance (ANOVA) followed by Tukey’s multiple comparisons test for comparisons among multiple groups. The number of biological replicates (n) is indicated in each figure legend. Statistical significance is indicated as *p < 0.05, **p < 0.01, and ***p < 0.001.

**Supplementary Fig 20.** Semi-quantitative analysis of uPAR immunofluorescence staining (n=3 biologically independent samples). Data are presented as mean ± standard error of the mean (SEM). Statistical analysis was performed using two-tailed Student’s t-test for comparisons between two groups and one-way analysis of variance (ANOVA) followed by Tukey’s multiple comparisons test for comparisons among multiple groups. The number of biological replicates (n) is indicated in each figure legend. Statistical significance is indicated as *p < 0.05, **p < 0.01, and ***p < 0.001.

**Supplementary Fig 21.** Agarose gel electrophoresis analysis of LNP@A versus empty LNP@A.

**Supplementary Fig 22.** Batch-to-batch characterization of LNP@A, including particle size, polydispersity index (PDI), zeta potential, and encapsulation efficiency (n=3). Data are presented as mean ± standard error of the mean (SEM). Statistical analysis was performed using two-tailed Student’s t-test for comparisons between two groups and one-way analysis of variance (ANOVA) followed by Tukey’s multiple comparisons test for comparisons among multiple groups. The number of biological replicates (n) is indicated in each figure legend. Statistical significance is indicated as *p < 0.05, **p < 0.01, and ***p < 0.001.

**Supplementary Fig 23. A.** Semi-quantitative analysis of live/dead staining in cells treated with LNP@A at different time points (n=3 biologically independent samples). **B.** qRT-PCR analysis of inflammatory cytokine expression in BMSCs treated with LNP@A formulations containing different ionizable lipid components (n=3 biologically independent samples). Data are presented as mean ± standard error of the mean (SEM). Statistical analysis was performed using two-tailed Student’s t-test for comparisons between two groups and one-way analysis of variance (ANOVA) followed by Tukey’s multiple comparisons test for comparisons among multiple groups. The number of biological replicates (n) is indicated in each figure legend. Statistical significance is indicated as *p < 0.05, **p < 0.01, and ***p < 0.001.

**Supplementary Fig 24.** Representative immunofluorescence images (scale bar = 10 μm) and semi-quantitative analysis of cellular uptake of LNP@A and scrambled peptide-modified LNP (LNP@SP) in senescent BMSCs (n=3 biologically independent samples). Data are presented as mean ± standard error of the mean (SEM). Statistical analysis was performed using two-tailed Student’s t-test for comparisons between two groups and one-way analysis of variance (ANOVA) followed by Tukey’s multiple comparisons test for comparisons among multiple groups. The number of biological replicates (n) is indicated in each figure legend. Statistical significance is indicated as *p < 0.05, **p < 0.01, and ***p < 0.001.

**Supplementary Fig 25.** Western blot analysis of the effects of LNP@A treatment on the subcellular distribution of ABCA1. Aged BMSCs were used in all groups except the young control group.

**Supplementary Fig 26. A.** Western blot analysis of mTORC1 signaling activity following LNP@A treatment. **B.** Representative confocal images of LC3II and TOM20 staining after LNP@A intervention (scale bar = 10μm). Colocalization analysis of LC3II and TOM20 staining (n=3 biologically independent samples). **C.** Representative confocal images of Lysotracker and Mitotracker after LNP@A intervention (scale bar = 10μm). Colocalization analysis of Lysotracker and Mitotracker staining (n=3 biologically independent samples). **D.** Representative fluorescence images of JC-1 mitochondrial membrane potential after LNP@A intervention (scale bar = 10μm). Semi-quantitative analysis of JC-1 mitochondrial membrane potential (n=3 biologically independent samples). Data are presented as mean ± standard error of the mean (SEM). Statistical analysis was performed using two-tailed Student’s t-test for comparisons between two groups and one-way analysis of variance (ANOVA) followed by Tukey’s multiple comparisons test for comparisons among multiple groups. The number of biological replicates (n) is indicated in each figure legend. Statistical significance is indicated as *p < 0.05, **p < 0.01, and ***p < 0.001.

**Supplementary Fig 27.** qRT-PCR analysis of osteogenic marker gene expression in BMSCs following LNP@A treatment (n=3 biologically independent samples). Data are presented as mean ± standard error of the mean (SEM). Statistical analysis was performed using two-tailed Student’s t-test for comparisons between two groups and one-way analysis of variance (ANOVA) followed by Tukey’s multiple comparisons test for comparisons among multiple groups. The number of biological replicates (n) is indicated in each figure legend. Statistical significance is indicated as *p < 0.05, **p < 0.01, and ***p < 0.001.

**Supplementary Fig 28.** Representative H&E staining images of major organs, including the heart, liver, spleen, lung, and kidney, showing no obvious histopathological abnormalities following LNP@A treatment (scale bar = 200μm).

**Supplementary Fig 29.** Assessment of total cholesterol levels and lysosomal cholesterol accumulation in bone tissues following LNP@A treatment (n=8 biologically independent samples). Data are presented as mean ± standard error of the mean (SEM). Statistical analysis was performed using two-tailed Student’s t-test for comparisons between two groups and one-way analysis of variance (ANOVA) followed by Tukey’s multiple comparisons test for comparisons among multiple groups. The number of biological replicates (n) is indicated in each figure legend. Statistical significance is indicated as *p < 0.05, **p < 0.01, and ***p < 0.001.

**Supplementary Fig 30. A.** Evaluation of locomotor function in rats by CatWalk gait analysis and hindlimb weight distribution assessment (n=8 biologically independent samples). **B.** Biomechanical assessment of the femoral condyle and newly formed bone by compression testing (n=8 biologically independent samples). Data are presented as mean ± standard error of the mean (SEM). Statistical analysis was performed using two-tailed Student’s t-test for comparisons between two groups and one-way analysis of variance (ANOVA) followed by Tukey’s multiple comparisons test for comparisons among multiple groups. The number of biological replicates (n) is indicated in each figure legend. Statistical significance is indicated as *p < 0.05, **p < 0.01, and ***p < 0.001.

**Table S1.** Primers used in real-time PCR

| Gene | Primer | Sequence |
| --- | --- | --- |
| RUNX2 | Forward primer | CAGTTCCTAACGGGCACCAT |
|  | Reverse primer | TTAGGGTCTCGGAGGGAAGG |
| OSX | Forward primer | GATGGCGTCCTCTCTGCTTG |
|  | Reverse primer | TTTCCCAGGGCTGTTGAGTC |
| COL I | Forward primer | CCAGCCGCAAAGAGTCTACAT |
|  | Reverse primer | GATGTCTTCTTGGCCATGCG |
| ALP | Forward primer | AAACTCGCTTATGGTCCCCG |
|  | Reverse primer | AATACCGGAAGGAGTGCTGC |
| OCN | Forward primer | GAATAGACTCCGGCGCTACC |
|  | Reverse primer | TCCTGGAAGCCAATGTGGTC |
| ABCA1 | Forward primer | GCAGCGACCATGAAAGTGAC |
|  | Reverse primer | GAGGCGGTCATCAATCTCGT |
| ABCG1 | Forward primer | GCCATCCCTGTCTTGCTCTT |
|  | Reverse primer | CCCTCGAAGCCGTATCTGAC |
| ABCA5 | Forward primer | GGAGTACCCCGTTCATTCAGG |
|  | Reverse primer | CTGTCCAATCCAGTAGGCGG |
| IL-1β | Forward primer | ATGCCTCGTGCTGTCTGACC |
|  | Reverse primer | TTTGTCGTTGCTTGTCTCTCCTTG |
| IL-6 | Forward primer | CTTCCAGCCAGTTGCCTTCTTG |
|  | Reverse primer | TGGTCTGTTGTGGGTGGTATCC |

**Table S2.** The sequence of shRNA and over expression

| Gene | Sequence |
| --- | --- |
| shABCA1 | GCCTTGTTAAGTATGGAGAAATCAAGAGTTTCTCCATACTTAACAAGGCTTTTTT |
| shABCG1 | CCAGTCCTTAGGCCTACTCATTCAAGAGATGAGTAGGCCTAAGGACTGGTTTTTT |
| shLYPLA1 | GGCATTCCTTCTAACAGAATTTCAAGAGAATTCTGTTAGAAGGAATGCCTTTTTT |
| shLYPLA2 | GGATTCCTGTGACACTCAACATCAAGAGTGTTGAGTGTCACAGGAATCCTTTTTT |
| shPPT1 | GCTTCTTCTTGAATGTCAACCTCAAGAGGGTTGACATTCAAGAAGAAGCTTTTTT |
| shABHD17A | CAGACAGTATCATCCTGTATGTCAAGAGCATACAGGATGATACTGTCTGTTTTTT |
| shABCA5 | CGACGTTCTAGTTACTGAAGATCAAGAGTCTTCAGTAACTAGAACGTCGTTTTTT |

OE-HA-APT2: ATGTGTGGTAACAACATGTCTGTGCCCCTGCTCACCGACGCAGCCACCGTGTCCGGAGCTGAGCGGGAAACGGCCGCGGTTATTTTTTTACATGGACTTGGAGACACAGGGCACAGCTGGGCTGACGCCCTCTCCACCATCCGGCTTCCTCATGTCAAGTACATCTGTCCCCATGCGCCCAGGATTCCTGTGACACTCAACATGAAGATGGTAATGCCCTCCTGGTTTGACCTGATGGGGCTGAGTCCAGATGCCCCAGAGGATGAAGCCGGCATCAAGAAGGCCGCAGAGAACATCAAGGCTTTGATTGAACACGAGATGAAGAACGGGATTCCTGCCAACCGAATCGTCCTGGGTGGCTTTTCGCAGGGTGGGGCCCTGTCCCTCTATACAGCACTTACCTGCCCCCACCCTCTGGCCGGCATCGTGGCTTTGAGCTGCTGGCTGCCTCTGCACCGGAACTTCCCCCAAGCAGCCAATGGCAGTGCCAAGGACCTGGCCATCCTTCAATGCCACGGCGAGCTGGACCCCATGGTACCTGTTCGGTTTGGGGCCCTGACAGCTGAGAAGCTCCGGACAGTTGTCACACCTGCCAGGGTCCAGTTTAAGACATACCCAGGTGTCATGCACAGCTCCTGTCCTCAGGAGATGGCAGCTGTAAAGGAATTTCTGGAGAAACTTCTGCCTCCGGTCGGTGGTGGCGGATCCTACCCATACGATGTTCCAGATTACGCTTAA

OE-HA-DHHC8: ATGCCCCGCAGCCCCGGGACGCGCCTCAAACCCGCCAAGTACATCCCGGTGGCCACGGCCGCGGCGCTGTTGGTTGGCTCCAGCACCCTCTTCTTCGTATTCACGTGCCCGTGGTTGACAAGAGCTGTCTCTCCAGCTATTCCTGTCTACAATGGCATCCTCTTCCTCTTTGTCCTGGCCAACTTCAGTATGGCTACCTTCATGGACCCCGGAGTCTTCCCCCGAGCGGACGAGGACGAGGACAAGGAGGATGACTTCCGGGCCCCACTGTACAAGAACGTGGATGTGCGGGGCATCCAGGTCCGCATGAAGTGGTGTGCAACATGCCACTTCTACCGTCCGCCGCGCTGCTCACACTGCAGCGTCTGCGACAACTGTGTGGAGGACTTTGACCACCACTGCCCCTGGGTGAACAACTGCATCGGACGCCGTAACTACCGTTACTTCTTCCTGTTCCTGCTGTCACTCAGCGCACACATGGTGGGGGTGGTGGCCTTCGGCCTGCTCTACGTGCTCAACCACTCGGAGGGGCTGGGGGCGGCCCACACCACCATCACCATGGCTGTCATGTGTGTGGCTGGCCTTTTCTTCATTCCTGTCATCGGCCTCACTGGCTTCCACGTGGTACTGGTCACCCGGGGACGCACCACCAATGAGCAGGTGACTGGGAAGTTCCGCGGGGGTGTGAATCCCTTCACCCGAGGCTGCTATGGGAACGTGGAGCACGTGTTATGCAGTCCCCTGGCGCCCCGGTATGTGGTGGAAGCTCCCAGGATGCCACTCTCAGTGAGCCTAAAGCCACCCTTCCTGAGGCCTGAGCTCCTGGAACGAGCTGTGCCCCTCAAGGTCAAACTCAGCGACAATGGGCTGAAAGCTGGCCGCAGCAAGTCTAAGGGCAGTCTAGACCAGCTGGATGAGAAACCTCTGGACCTGGGACCTCCACTGCCCCCCAAGATGGAGGCTGGCACCTTTGGAAGAGATCTGAAGACCCCAAGACCTGGCAGTGCTGAGAGTGCCCTGTCAGTACAGAGGACCAGCCCCCCAACACCTGCCATGTATAAGTTCCGGCCAGCCTTCTCCACTGCTCCCAAGACACCCTTTTGTGGACCTAGTGAGCAGGTCCCAGGCCCTGACTCCCTCACTCTGGCCGATGACAGCACCCACAGTCTAGACTTTGTATCAGAGCCCAGCCTGGATCTCCCAGACCATGGGCCTGGTGGTCTGCATCCTACCTACCCTCCCTCCCCACCCCTCAGTGCCACTGATGCCTTCTCAGGTGCCTTACGCTCCCTGAGTCTCAAGGCTGCCAGTCGGCGGGGTGGGGACCACATGACCTTACAGCCACTGCGCTCTGAAGGTGGGCCCCCTACACCCCACCGTGGTCTCTTTGCTCCTCACGCACTGCCCAACCGAAATGGCAGCCTGTCATACGACAGCCTACTTAACCCTGGCTCACCCAGTGGCCACGCATGCCCCACACACCCCTCTGTTGGTATGGCCGGCTACCATTCACCCTACCTGCACCCTGGGCCATCAGATCCTCCACGGCCCCCACCCCGCAGCTTCAGCCCTGTGCTGGGTCCCCGGCCTAGGGAGCCCTCTCCTGTGCGCTATGACAACCTGTCTCGGACCATCATGGCCTCTATCCAGGAGCGCAAGGACAGAGAAGAGCGTGAACGGCTGCTGCGGTCCCAGACTGACTCACTCTTTGGCGACTCTGGTGTCTATGACACACCCAGCTCCTACAGCCTGCAACAGGCCAGTGTGTTAACAGAAGGTCCCCGCGGCTCTGTGCTGCGCTATGGCTCCAGGGATGACCTCGTGGCCGGGCCTGGCTTTGGTGGTGCCCGAAATCCTGCCCTGCAGACATCATTGTCCTCACTGTCCAGCTCCATGAGTCGGGCACCTCGGACATCCTCCTCTTCCCTGCAGGCTGACCAGGCCAACAACAATGCCCCAGGACCCCGGCCTGGCAGTGGTTCACACAGGTCACCTGCCCGTCAGGGCCTGCCTTCCCCACCAGGCACCCCCCGATCGCCCTCCTACTCGGGCTCCAAGGCTGTCGCCTTCATCCACACGGACCTTCCGGACCGGCAGCCCTCACTGGCTATGCAGAGGGATCACCCTCAGCTGAAGACCCCCCCAAGTAAGCTTAACGGTCAGTCCCCGGGCATGGCCCGGCTGGGGCCTGCTGCCAGCCCCATGGGGCCCAACGCCAGCCCTGCCCGGCACACGCTGGTTAAGAAGGTGTCCGGCGTGGGTGGGACTACGTACGAAATCTCGGTGGGTGGTGGCGGATCCTACCCATACGATGTTCCAGATTACGCTTAA

OE-HA-RA: ATGTGTGGAAACAATATGTCTGTTCCTTTGCTGACCGATGCTGCTACCGTGAGCGGGGCTGAGCGGGAAACTGCTGCAGTCATTTTTCTCCACGGACTGGGCGACACCGGACATAGTTGGGCTGACGCACTCAGTACAATCAGACTCCCACATGTTAAGTACATCTGCCCCCACGCCCCACGCATCCCTGTCACCCTGAATATGAAGATGGTGATGCCCTCTTGGTTTGACCTGATGGGCCTCAGCCCAGATGCACCTGAGGACGAAGCCGGAATCAAAAAGGCCGCCGAGAACATCAAGGCTCTGATAGAACACGAGATGAAAAATGGAATTCCTGCAAATAGGATTGTGCTTGGTGGCTTTTCCCAGGGTGGCGCTCTGTCACTCTACACAGCCTTGACCTGCCCTCACCCACTGGCCGGTATCGTGGCCCTCAGTTGCTGGCTTCCACTGCACAGGAATTTCCCTCAAGCAGCTAATGGGTCTGCAAAAGACTTGGCCATCCTGCAGTGCCACGGCGAGTTGGACCCCATGGTCCCTGTAAGGTTCGGGGCTCTCACTGCTGAGAAACTGCGGACTGTCGTGACTCCCGCACGCGTGCAGTTTAAGACCTACCCTGGAGTGATGCACTCTTCTTGCCCTCAAGAAATGGCCGCCGTTAAGGAGTTTCTTGAGAAACTGCTGCCTCCCGTGGGGGGCGGCTCCGGCGGAGGCTCAGGAGGGGGCAGCATGACTTCACGGAAGAAAGTGCTCCTCAAGGTCATCATCCTCGGTGACTCCGGGGTAGGAAAGACCTCCTTGATGAATCAGTACGTGAACAAAAAGTTCTCCAATCAGTATAAGGCAACCATCGGGGCCGATTTTTTGACCAAGGAGGTCATGGTGGATGACAGGCTGGTCACCATGCAGATCTGGGACACTGCTGGCCAGGAGCGCTTTCAATCACTTGGAGTGGCATTTTACCGCGGGGCCGACTGCTGTGTACTGGTGTTCGATGTAACTGCCCCAAATACTTTCAAGACTTTGGACTCCTGGCGGGACGAATTCCTGATCCAGGCTAGTCCCCGAGACCCGGAGAATTTCCCCTTCGTGGTTCTCGGTAATAAGATCGATCTGGAGAACCGACAGGTAGCTACCAAGCGGGCCCAGGCATGGTGTTACTCCAAGAACAATATCCCTTACTTCGAAACATCTGCAAAGGAAGCCATTAATGTGGAACAGGCCTTCCAGACCATCGCCAGAAACGCCCTGAAGCAGGAGACAGAGGTTGAGCTGTATAACGAGTTCCCTGAACCCATTAAGCTGGACAAGAATGAGAGGGCCAAGGCATCTGCTGAATCATGTTCATGTGGTGGTGGCGGATCCTACCCATACGATGTTCCAGATTACGCTTAA

ABCA1.Res: ATGGCTTGTTGGTCTCAGTTAAGGTTGCTGCTGTGGAAGAATCTGACATTTCGAAGAAGACAAACATGTCAGCTGTTACTGGAAGTGGCCTGGCCTCTCTTTATCTTCCTGATCCTGATTTCTGTCCGCCTGAGCTACCCACCCTATGAACAACATGAGTGTCACTTTCCAAATAAAGCCATGCCTTCTGCAGGAACCCTTCCCTGGGTCCAGGGGATTATCTGTAATGCCAATAACCCTTGCTTCCGTTATCCAACTCCAGGCGAGGCTCCCGGTGTTGTTGGAAACTTTAACAAGTCCATTGTGTCCCGCCTATTCTCAGATGCTCAACGGATTCTTTTGTACAGCCAAAAGGACACCAGCATTAGGGACATGCACAAGGTCCTGAGAACATTACAGCAGATCAAGCATCCCAACTCAAATTTGAAGCTTCAGGATTTCCTGGTGGACAATGAAACCTTCTCTGGATTCCTACAGCACAGTTTGTCCCTTCCAAGGTCCGCTGTGGACAATCTGCTACAGGCTGATGTCAGTCTCCAGAAGGTGTTTTTGCAAGGCTACCAGTTACATTTGGCCAGTCTGTGTAATGGATCAAAATTAGAAGAAATCATTCGGCTTGGAGATCTGGAAGTTTCTGCCCTTTGTAGTCTACCTAGGGAGAAGCTAGATGCAGCAGAGCGCGAGCTGCGTTCTAACATGGACATCCTGAAGCCAGTCATGACAAAACTAAACTCCACGTCTCTTCTCCCGACCCAGCATCTGGCTGAAGCCACCACAACGTTGCTTGACAGCTTAGGGGGGCTGGCCCAAGAGCTCTTCAGCACAAAGAGCTGGAGCGACATGCGGCAGGAGGTGATGTTTCTGACCAACGTGAACAACTCCGGCTCCTCCACCCAGATATACCAGGCTGTGTCCCGAATCGTCTGTGGTCACCCAGAGGGCGGGGGCCTGAAGATCAAGTCCCTCAACTGGTACGAGGATAACAACTACAAAGCCCTCTTTGGAGGGAATGGCACCGAGGAAGACACAGACACCTTCTATGACAATTCTACAACTCCTTATTGCAATGATTTGATGAAGAACTTGGAGTCTAGTCCTCTTTCTCGAATTATTTGGAAGGCACTCAAGCCACTGCTCATTGGGAAGATTCTGTATACACCTGACACACCAGCTACAAGGCAGGTCATGGCTGAGGTGAACAAGACCTTTCAGGAGCTGGCTTTGTTCCATGACCTGGAGGGCATGTGGGAAGAACTCAGCCCCCAAATTTGGACCTTCATGGAGAGCAGCCAAGAGATGGACCTTGTGCGGACGATGTTGGACAGCAGAGGCAATGACCAGTTTTGGGAACGAAAGTTGGATGGATTAGATTGGACTGCCCAAGACATCATGGCATTTCTGGCCAAGAACCCAGAGGATGTTCAGTCCCCAAATGGCTCTGTGTATACATGGAGAGAAGCTTTCAATGAGACCAACCAGGCAATCCAGACGATATCTCGATTCATGGAGTGTGTCAACCTGAACAAGCTGGAGCCCATCCCAACAGAAGTCAGGCTCATCAACAAGTCCATGGAGCTGCTGGATGAGAGGAAGTTCTGGGCTGGTATCGTGTTCACAGGAATCACTCCTGATAGTGTGGAGCTGCCCCACCATGTAAAGTACAAGATCCGGATGGACATTGACAATGTTGAGAGAACCAATAAGATCAAGGATGGGTACTGGGACCCTGGTCCCCGGGCTGACCCTTTTGAAGATATGCGCTATGTCTGGGGTGGCTTCGCCTACTTGCAGGATGTGGTGGAGCAGGCGATCATCAGAGTGCTGACGGGGACTGAGAAGAAAACGGGCGTCTATGTGCAACAGATGCCCTACCCCTGTTATGTTGATGACATTTTTCTGCGGGTCATGAGCCGCTCAATGCCCCTCTTCATGACTCTGGCCTGGATCTACTCTGTGGCTGTGATCATCAAGAGCATTGTGTATGAGAAGGAGGCTCGGTTGAAGGAGACCATGCGCATCATGGGCCTGGACAATGGCATCCTCTGGTTTAGCTGGTTCATTAGTAGTCTCATCCCTCTGCTCGTGAGCGCTGGCCTGTTGGTGATCATCTTGAAGTTAGGAGATCTGCTGCCCTATAGTGACCCCAGCGTGGTGTTCGTCTTCCTCTCTGTGTTTGCCGTGGTGACCATTCTACAGTGCTTCCTCATTAGCACACTCTTCTCCCGGACCAACCTGGCAGCAGCCTGTGGGGGCATCATCTACTTCACGCTGTACCTGCCCTATGTGCTGTGTGTAGCATGGCAGGACTATGTGGGCTTCTCCATCAAGATCTTTGCTAGCCTGCTGTCTCCTGTGGCTTTTGGATTCGGCTGTGAGTATTTTGCTCTTTTTGAGGAGCAAGGCATCGGGGTCCAATGGGACAATCTCTTTAAGAGCCCAGTGGAGGAGGATGGCTTCAATCTTACCACCTCAGTGTCCATGATGCTGTTTGACACCTTCATCTATGGCGTGATGACATGGTACATCGAAGCCGTCTTCCCAGGACAGTACGGAATTCCCAGGCCCTGGTATTTTCCTTGTACCAAGTCCTACTGGTTTGGGGAGGAAATTGATGAGAAGAGCCACCCTGGTTCCAGCCAGAAGGGTGCGTCAGAAATCTGCATGGAAGAGGAGCCCACTCACCTGAAGCTGGGGGTATCCATTCAGAACCTGGTGAAGGTTTACCGAGATGGCATGAAGGTTGCTGTGGATGGTTTGGCGCTAAATTTTTACGAGGGCCAGATCACCTCCTTCCTGGGCCACAATGGAGCAGGGAAGACCACCACCATGTCAATACTGACTGGGTTGTTTCCCCCAACTTCTGGCACTGCCTACATCCTGGGGAAGGACATTCGATCTGAGATGAACTCTATCCGGCAGAACCTGGGAGTCTGTCCCCAGCATAATGTGTTGTTTGACATGCTGACAGTCGAAGAGCACATCTGGTTCTATGCCCGCCTAAAGGGGCTCTCAGAGAAGCATGTGAAAGCAGAGATGGAGCAGATGGCCCTGGATGTTGGCTTACCCCCCAGCAAGCTGAAAAGCAAAACAAGTCAGCTCTCTGGTGGGATGCAGAGAAAGCTGTCTGTGGCCTTGGCCTTCGTGGGTGGATCCAAGGTTGTCATTCTGGATGAGCCCACAGCAGGTGTGGACCCATACTCTCGCAGGGGAATATGGGAGCTCCTGCTGAAATACCGACAAGGCCGCACCATTATTTTGTCTACACACCACATGGATGAAGCAGACATCCTAGGGGACAGAATTGCCATCATTTCCCATGGGAAGCTGTGTTGTGTGGGCTCCTCCCTGTTTTTGAAAAACCAGTTGGGAACGGGTTACTACCTGACCCTGGTCAAGAAAGATGTGGAATCGTCCCTCAGTTCCTGCAGAAACAGTAGCAGTACTGTGTCTTGTCTGAAAAAGGAGGACAGTGTTTCTCAGAGCAGTTCTGATGCTGGCCTGGGCAGCGACCATGAAAGTGACACGCTGACCATCGATGTCTCTGCTATCTCCAACCTCATCAGGAAACATGTGTCCGAAGCCCGGCTGGTGGAGGACATTGGGCACGAACTGACCTATGTGCTGCCGTACGAAGCTGCTAAGGAGGGAGCCTTTGTGGAGCTCTTCCACGAGATTGATGACCGCCTCTCAGACCTGGGCATCTCCAGTTACGGCATCTCAGAGACCACCCTAGAAGAAATATTCCTCAAAGTGGCTGAAGAGAGTGGGGTGGATGCTGAGACCTCAGACGGTACTTTGCCAGCAAGACGAAACAGACGGGCCTTCGGTGACAAGCAGAGCTGTCTGCACCCATTTACAGAGGACGATGCTGTGGATCCGAATGACTCTGACCTAGACCCAGAATCCAGGGAGACAGACCTGCTCAGCGGGATGGATGGCAAAGGCTCCTACCAGCTAAAGGGCTGGAAACTCACCCAGCAGCAGTTTGTGGCCCTCTTGTGGAAAAGGCTGCTGATTGCCCGGCGGAGTAGAAAGGGTTTCTTTGCTCAGATTGTCCTGCCGGCTGTCTTTGTTTGCATCGCCCTGGTCTTCAGCCTGATCGTGCCACCCTTTGGCAAGTACCCCAACCTGGAACTTCAACCCTGGATGTATAACGAGCAGTATACATTTGTCAGTAACGATGCTCCAGAGGACATGGGCACCCAGGAACTCCTCAATGCTCTAACCAAAGATCCAGGCTTCGGGACCCGTTGTATGGAAGGAAACCCAATCCCAAACACTCCTTGCTTGGTCGGGGAGGAGGACTGGACCACTGGCCCAGTTCCCCAGACCCTCATGGACCTCTTCCAAAATGGGAACTGGACGATGAAGAACCCCTCACCTTCGTGCCAGTGTAGCAGTGACAAAATCAAGAAGATGCTGCCTGTGTGTCCTCCAGGGGCAGGGGGGCTGCCGCCTCCTCAGAGAAAACAGAAGACTGCAGACATCCTTCAGAATCTGACCGGAAGAAATATTTCAGATTACCTGGTGAAGACATATGTGCAGATCATAGCAAAAAGCTTAAAGAATAAGGTCTGGGTGAATGAGTTTCGGTATGGCGGGTTTTCCCTGGGTGTCAGTGATTCTCAAGCACTTCCTCCGAGCCAAGAAGTTAATAATGCTATCAAGCAAATGAAGAAACTCCTGAAGCTGACCAAGGACAGCTCTGCAGATCGGTTCCTCAGCAGCTTGGGAAGGTTCATGACAGGACTGGACACCAAAAACAATGTCAAGGTGTGGTTCAATAACAAGGGCTGGCATGCCATCAGTTCATTCCTGAATGTCATCAACAATGCCATTCTCCGGGCCAACCTGCAGAAGGGAGAGAACCCAAGCCAGTATGGGATTACTGCTTTCAACCATCCCCTGAACCTCACCAAGCAGCAGCTCTCGGAGGTGGCTCTGATGACCACATCTGTCGACGTCCTTGTGTCCATCTGTGTCATCTTTGCAATGTCCTTTGTGCCAGCCAGCTTTGTTGTGTTCCTGATCCAGGAGCGTGTGAGCAAAGCCAAACACCTTCAGTTCATCTGCGGCGTGAAGCCTGTCATCTACTGGCTCTCCAACTTTGTCTGGGATATGTGTAATTATGTGGTCCCTGCTACACTGGTCGTTATCATCTTCATCTGCTTCCAGCAGAAGTCCTATGTATCCTCCACCAACCTGCCTGTTCTAGCCCTTCTGCTCTTGCTATATGGGTGGTCGATCACTCCTCTCATGTACCCAGCGTCCTTTGTGTTCAAGATCCCCAGCACTGCCTACGTGGTCCTCACCAGTGTGAACCTCTTCATCGGCATCAATGGTAGTGTGGCCACTTTCGTCCTGGAGCTCTTTACAAACAATAAATTCAATGACATCAATGACATCCTGAAATCTGTGTTTCTTATTTTCCCACATTTTTGCCTGGGGCGAGGGCTTATTGACATGGTGAAAAACCAGGCGATGGCTGATGCCCTGGAGAGGTTTGGGGAGAACCGCTTCGTCTCTCCACTCTCTTGGGACTTAGTAGGACGGAACCTTTTTGCCATGGCCGTGGAAGGGGTGGTGTTCTTCCTCGTTACGGTTCTAATCCAGTACCGATTTTTCATCAGGCCCAGACCTGTAAAGGCGAGGCTTCCTCCTTTGAATGACGAGGACGAGGACGTGAGGAGGGAGAGACAGAGGATTCTGGAGGGTGGAGGACAGAATGACATCCTAGAGATCAAAGAACTGACCAAGATCTACAGAAGGAAGCGGAAGCCTGCAGTGGACAGAATCTGCGTTGGCATCCCTCCTGGAGAGTGCTTTGGACTCCTGGGAGTTAATGGAGCTGGGAAGACATCAACATTCAAGATGCTGACTGGAGATACCGCTGTGACCAGAGGGGATGCTCTTCTTAACAAGAACAGCATCTTATCAAACATCCATGAAGTACATCAAAACATGGGCTACTGCCCTCAGTTCGATGCCATCACGGAGCTTCTGACTGGAAGAGAGCATCTGGAGTTCTTTGCCCTTCTCAGGGGAGTCCCAGAAAAAGAAGTTGGCAAGGTTGGTGAATGGGCAATTCGCAAACTGGGACTCGTGAAATACGGCGAGAAATACGCCAGCAACTACAGTGGCGGTAACAAGCGGAAGCTTTCCACAGCCATTGCTCTGATTGGCGGGCCTCCTGTGGTGTTTCTGGATGAACCAACCACAGGCATGGACCCCAAAGCCCGGAGATTCCTGTGGAATTGTGCCCTAAGCATTATCAAGGAGGGAAGATCCGTAGTTCTTACATCTCATAGCATGGAGGAATGTGAAGCTCTTTGTACCAGGATGGCCATAATGGTCAATGGGAGGTTCAGGTGCCTCGGCAGTGTCCAACATCTGAAAAACAGGTTTGGAGATGGTTATACCATAGTTGTACGAATAGCGGGCTCCAACCCTGACCTAAAGCCTGTCCAGGAGTTCTTTGGACTTGCCTTTCCTGGAAGTGTTCTGAAAGAGAAACATCGAAACATGCTACAGTACCAGCTTCCGTCCTCCTTGTCATCTCTGGCCAGGATATTCAGCATTCTCTCCCAGAGCAAAAAGCGACTCCACATAGAAGACTACTCTGTCTCTCAGACAACACTTGACCAAGTATTCGTGAACTTTGCCAAGGACCAAAGTGATGATGACCACTTAAAGGACCTGTCACTACACAAAAACCAGACAGTGGTGGATGTGGCTGTTCTCACATCCTTCCTGCAGGACGAGAAAGTGAAAGAAAGTTACGTAGGTGGTGGCGGATCCTACCCATACGATGTTCCAGATTACGCTTAA

**Table S3.** The sequence of RA mRNA

RA mRNA sequence: m7GAGGAAAUAAGAGAGAAAACUCUUCUGGUCCCCACAGACUCAGAGAGAACCCGCCACCAUGUGUGGGAAUAAUAUGUCUGUGCCUCUACUAACUGAUGCUGCCACGGUCAGCGGCGCAGAGCGCGAAACCGCUGCUGUCAUCUUCCUGCACGGAUUAGGUGACACUGGCCAUUCCUGGGCUGAUGCGCUCAGUACCAUCCGGCUCCCCCAUGUCAAGUACAUCUGCCCCCAUGCCCCAAGAAUCCCUGUGACACUUAACAUGAAGAUGGUAAUGCCCAGCUGGUUUGACCUGAUGGGCCUGUCCCCAGACGCCCCUGAGGAUGAGGCUGGUAUUAAGAAAGCCGCCGAGAACAUUAAAGCUCUAAUUGAGCAUGAAAUGAAAAAUGGCAUACCAGCCAAUAGGAUAGUACUGGGAGGCUUCAGUCAGGGCGGGGCACUGUCUCUUUAUACAGCCCUCACCUGUCCCCACCCUCUUGCUGGGAUUGUGGCACUCAGCUGCUGGCUGCCGUUGCACAGGAACUUUCCUCAGGCUGCAAACGGGAGCGCAAAGGACCUUGCUAUUCUGCAGUGCCACGGAGAGCUGGACCCUAUGGUUCCCGUGCGCUUCGGGGCGUUGACAGCAGAAAAGCUUAGGACAGUGGUGACUCCUGCCCGCGUGCAGUUUAAAACUUACCCAGGAGUGAUGCACUCUUCGUGUCCACAGGAGAUGGCGGCCGUUAAAGAAUUUCUGGAGAAACUCCUCCCACCUGUAGGGGGAGGGUCAGGAGGUGGCUCCGGCGGUGGUAGUAUGACCAGCCGUAAGAAGGUGCUGCUGAAGGUCAUAUUGGGUGACAGUGGCGUUGGAAAAACCUCAUUGAUGAAUCAGUAUGUAAAUAAAAAGUUCAGCAACCAGUACAAGGCCACCAUCGGCGCUGACUUUUUGACUAAGGAAGUUAUGGUGGAUGACCGAUUAGUUACCAUGCAGAUCUGGGACACUGCAGGCCAGGAAAGAUUCCAGAGUUUAGGUGUCGCCUUCUAUAGAGGAGCAGACUGCUGUGUGCUGGUGUUUGAUGUCACAGCCCCCAAUACAUUCAAGACAUUGGAUUCUUGGAGGGAUGAAUUCUUGAUCCAGGCUAGCCCGCGAGAUCCAGAAAACUUCCCUUUUGUCGUUCUGGGGAACAAGAUCGAUCUGGAAAAUCGGCAGGUGGCAACGAAACGGGCCCAAGCCUGGUGCUACUCUAAGAACAACAUUCCCUAUUUUGAGACCUCCGCGAAGGAGGCCAUCAAUGUUGAGCAAGCUUUCCAAACAAUUGCAAGAAACGCCCUAAAGCAAGAAACGGAGGUGGAGCUCUACAACGAAUUUCCGGAGCCAAUCAAACUGGACAAGAACGAGAGAGCAAAAGCUUCAGCUGAGUCAUGCUCCUGUUGAGCUGGAGCCUCGGUGGCCAUGCUUCUUGCCCCUUGGGCCUCCCCCCAGCCCCUCCUCCCCUUCCUGCACCCGUACCCCCGUGGUCUUUGAAUAAAGUCUGAGUGGGCGGCAUCUAGAAAAAAAAAAAAAAAAAAAAAAAAAAAAAAAAAAAAAAAAAAAAAAAAAAAAAAAAAAAAAAAAAAAAAAAAAAAAAAAAAAAAAAAAAAAAAAAAAAAA
